# Effectiveness of text messages in the adherence to COVID-19 prevention practices: a quasi-experimental study*

**DOI:** 10.1590/1518-8345.7810.4707

**Published:** 2025-12-01

**Authors:** Vinicius Lino de Souza, Sheila Coelho Ramalho Vasconcelos Morais, Rafaela Batista dos Santos Pedrosa, Mirian Ueda Yamaguchi, Eduesley Santana Santos, Agueda Maria Ruiz Zimmer Cavalcante, Vinícius Batista Santos, Juliana de Lima Lopes

**Affiliations:** 1 Universidade Federal de São Paulo, Escola Paulista de Enfermagem, São Paulo, SP, Brazil. Universidade Federal de São Paulo Escola Paulista de Enfermagem SP São Paulo Brazil; 2 Scholarship holder at the Conselho Nacional de Desenvolvimento Científico e Tecnológico (CNPq), Brazil. Scholarship holder at the Conselho Nacional de Desenvolvimento Científico e Tecnológico Brazil; 3 Universidade Federal do Pernambuco, Departamento de Enfermagem, Recife, PE, Brazil. Universidade Federal do Pernambuco Departamento de Enfermagem PE Recife Brazil; 4 Universidade Estadual de Campinas, Departamento de Enfermagem, Campinas, SP, Brazil. Universidade Estadual de Campinas Departamento de Enfermagem SP Campinas Brazil; 5 Centro Universitário de Maringá, Departamento de Saúde, Maringá, PR, Brazil. Centro Universitário de Maringá Departamento de Saúde PR Maringá Brazil; 6 Universidade Federal de Sergipe, Departamento de Enfermagem, Maceió, SE, Brazil. Universidade Federal de Sergipe Departamento de Enfermagem SE Maceió Brazil; 7 Universidade Federal do Goiás, Faculdade de Enfermagem, Goiânia, GO, Brazil. Universidade Federal do Goiás Faculdade de Enfermagem GO Goiânia Brazil

**Keywords:** Text Messaging, Coronavirus, Social Isolation, Masks, Treatment Adherence and Compliance, Behavior Therapy

## Abstract

to analyze the effectiveness of messages sent by cell phone on adherence to the use of masks, hand hygiene and social distancing during the COVID-19 pandemic.

a multicenter quasi-experimental clinical trial conducted in four Brazilian universities between July and October 2020. The sample consisted of 429 people from different Brazilian cities who received messages every two days for seven weeks about individual protection measures. Adherence to the measures was analyzed by an instrument developed by the researchers of the main center and refined in collaboration with researchers from the other centers, consisting of 13 questions and a five-point Likert scale, with the overall score ranging from 13 to 65 points, in which the higher the score, the greater the adherence. Statistical analysis was performed using multivariate analysis of variance (MANOVA), considering *p*<0.05 as significant.

an improvement in the adherence score was observed over the weeks. When compared with the score obtained before the intervention, there was a significant improvement in adherence in the last two weeks (*p*<0.001).

the intervention using messages sent by cell phone showed to be effective in promoting adherence to individual protection practices against COVID-19. However, the observed sample loss may have affected the results, limiting the generalizability of the findings. Therefore, the effects identified should be interpreted with caution. The Brazilian Registry of Clinical Trials (RBR-2s94rb).

## Highlights

**(1)** Use of low-cost technology to promote health education. **(2)** Strategy to strengthen or improve health policies. **(3)** Nursing’s leading role as an articulator of the adherence strategy.

## Introduction

COVID-19, caused by SARS-CoV-2, was identified as a global health problem and declared a pandemic by the World Health Organization (WHO) on March 11, 2020^(1)^. Although COVID-19 is no longer classified as a pandemic, cases of hospitalization and deaths related to the disease are still observed^(2-3)^.

At the beginning of the pandemic, governments in several countries adopted a series of strategies to contain the spread of SARS-CoV-2 and mitigate its impacts on health and the economy, such as community testing, contact screening, isolation, and individual protection measures^(4)^.

The recommendations for personal protection were related to the correct use and removal of face masks, a social distance of one meter between people and hand hygiene with soap and water or 70% alcohol^(5-6)^. These measures were not common practices among the Brazilian population, which posed an even greater challenge for the country’s governments. It has been suggested that, due to the high transmissibility of the virus and the lack of drug treatment and vaccination against COVID-19 at the beginning of the pandemic, controlling the disease would only be possible through these massive and rapid behavioral changes^(7)^.

Another challenge at the beginning of the pandemic was the shortage of surgical masks, where the WHO recommended that these masks be reserved for healthcare professionals, people with symptoms of the disease and caregivers or those who shared the same space with people with suspected COVID-19 or with respiratory symptoms^(5)^.

The WHO also stated that decision makers in each country should advise on the use of non-surgical masks regarding the number of layers and types of fabric and the shape and fit of the mask^(5)^. In view of these recommendations, the Brazilian National Health Surveillance Agency (ANVISA) recommended that non-surgical masks should consist of three layers of fabric, the outer layer made of non-waterproof fabric, the middle layer of breathable fabric and the inner layer of cotton fabric^(6)^.

Despite the significant lifestyle changes required, some studies have identified that the population wanted to practice individual protection measures against COVID-19 and that compliance with such recommendations was greater when associated with greater knowledge^(8-9)^.

A cross-sectional study conducted in Saudi Arabia with 502 older adult participants aged 65 years of age and above showed that more than half of the participants (52.2%) intended to practice individual protection measures against COVID-19. Moreover, the chances of practicing the preventive recommendations were 1.59 times (95% CI= 1.01-2.52) in women when compared to men and 2.72 times (95% CI= 1.44-5.16) higher in individuals with high level education^(8)^.

Similar results were found in the United States, where a survey of 506 residents revealed that participants who perceived the health practices recommended by the US Centers for Disease Control and Prevention (CDC) as effective had stronger intentions to engage in these practices. In addition, participants’ perception of the severity of COVID-19 in the United States was a significant factor (β = 0.48; *p* ≤ 0.001; 95% CI = 0.418–0.539). The perceived effectiveness of the recommended practices was responsible for the greatest variation in behavioral intentions (β = 0.19; *p*≤0.001; 95% CI=0.112–0.260)^(9)^.

In this sense, health education interventions to improve the population’s knowledge and adherence to individual protection measures have become essential and fundamental for disease control. Health education actions are a daily practice performed by nurses and several strategies can be adopted such as sending messages^(10-11)^.

A meta-analysis that evaluated nine randomized clinical trials with 1,121 participants showed a favorable impact of messages sent by cell phone when compared to usual care in medication adherence among individuals with type-2 diabetes mellitus (standardized mean difference: 0.36; 95% CI: 0.14-0.59)^(12)^.

A randomized clinical trial assessed whether behavioural nudges delivered through text messages could accelerate adherence to a health system’s COVID-19 vaccination policy among 2,000 health system employees in the Midwest and Southern regions of the US who were not adherent to the vaccination policy one month prior to its deadline. The results revealed that by the end of the two-week intervention, 363 participants in the text message nudge group (36.3%) and 318 participants in the control group (31.8%) had adhered to the vaccination policy, representing a significant increase of 4.9 percentage points (95% CI, 0.8 to 9.1, *p*=0.002) in adjusted analyses comparing the nudge group with the control group. Among participants who became adherent by the end of the 4-week follow-up period, the text message nudge significantly reduced the time to adherence by a mean of 2.4 days (95% CI, 2.1 to 4.7, *p*<0.001) and a median of 5.0 days (95% CI, 2.5 to 7.7, *p*<0.001) compared to the control group^(13)^.

These results are in line with another meta-analysis that analyzed the effect of messages for controlling excess weight and included 12 randomized controlled trials. Ten of the included studies showed a significant effect on participants’ weight loss and the pooled mean difference in body mass index change after the intervention was -0.43 kg/m² (95% CI -0.63 to -0.23 kg/m²)^(14)^.

In Brazil, text messaging is a widely used means of communication, especially in regions where Internet access is limited or where the use of advanced smartphones is not yet widespread^(15)^. This resource is especially valuable in public health contexts, as it facilitates the rapid dissemination of information and reaches diverse segments of the population, including those with lower levels of education or limited access to other sources of information^(15)^. In addition, cultural factors, such as trust in direct communication, can affect the way messages are received and the degree of adherence to guidance^(16)^.

While there exists research on public health campaigns and persuasion strategies, evidence on how these messages specifically influence populations during large-scale health crises, such as the COVID-19 pandemic, remains limited. Furthermore, while various persuasive approaches, including pro-social messaging, social proof, and moral appeals, are documented in the literature, few studies have explored the impact of these strategies in the context of emerging infectious diseases, especially considering the politicization that has marked the COVID-19 pandemic. Therefore, it is essential to investigate how this strategy has affected adherence to preventive behaviors in such a unique context.

Therefore, the aim of this study is to analyze the effectiveness of messages sent by cell phone on adherence to the use of masks, hand hygiene and social distancing during the COVID-19 pandemic.

## Method

### Design

This is a quasi-experimental, multicenter clinical trial conducted in accordance with the Transparent Reporting of Evaluations with Nonrandomized Designs (TREND) guidelines.

### Setting

This study was carried out at four public universities in the Brazilian states of São Paulo, Paraná, Pernambuco, and Sergipe.

### Period

Data were collected between July and October 2020.

### Population and selection criteria

The study population consisted of Brazilian adults. Eligibility criteria included: residing in Brazil, being 18 years of age or older, having a cell phone and Internet access to use the WhatsApp^®^ app, not having visual and/or cognitive impairments, being literate and proficient in Portuguese.

### Sample size determination

The sample size was calculated using G Power^®^ software, version 3.1.9.2 (available at https://www.gpower.hhu.de/). Based on a Cohen’s effect size of 0.15, a test power of 0.80 and a significance level of 5%, the minimum sample required was determined to be 351 participants. To compensate for possible losses during monitoring, 20% was added to the sample size, resulting in a minimum size of 421 participants.

### Interventions

The intervention consisted of sending previously validated messages^(17)^. The content of the text messages was developed through a narrative review of the literature, with the goal of analyzing recommendations related to mask use, hand hygiene, and social distancing during the SARS-CoV-2 pandemic.

Eighteen messages were created, covering topics such as disease, transmission methods, and preventive measures related to social distancing, mask use, and hand hygiene. These messages were crafted using concise sentences and an action-oriented tone to promote the adoption of COVID-19 preventive measures, accompanied by illustrative images relevant to each topic^(17)^. Here is an example of the text from one of the messages developed: “*The mask is a protective measure for the coronavirus, and you should always wear it when you leave home*”^(17)^.

Following their development, the messages were evaluated by a group of experts, who assessed them based on theoretical relevance, clarity, practical applicability, and vocabulary. Content validity was verified by calculating the Content Validity Index (CVI), with two rounds of evaluation required for the messages to achieve a CVI exceeding 90%, ensuring their validity^(17)^.

These messages were sent by cell phone two to three times per week, with a two-day interval between each sending, for seven weeks. The decision to use a two-day interval was informed by a previous study that examined the impact of sending text messages at this frequency, focusing on adherence and self-care in individuals with coronary artery disease. In that study, the majority of participants reported being satisfied with receiving messages two days apart^(18)^. There is no consensus in the literature on the ideal frequency for sending messages, with intervals ranging from daily to weekly or fortnightly^(12,19)^. The seven-week period for the intervention was adopted based on studies that used this health education strategy with other populations and that employed similar monitoring periods^(20-21)^.

### Variables and instruments

Adherence was assessed during an instrument initially developed by researchers at the main study center and later refined by eight health professionals with a master’s degree or doctorate in communicable or infectious diseases. Each expert had at least three years’ experience in communicable or infectious diseases or in managing situations related to pandemics. The refinement process was conducted via a Zoom meeting moderated by the study supervisor. During the session, each item of the instrument was carefully reviewed, with continuous discussions until the experts reached a consensus.

This instrument, which is in line with the messages used in the intervention, contains 13 questions on the correct use of face masks, the importance of hand hygiene and social distancing. Each item of the instrument has a five-point Likert scale (1= Never, 2= Rarely, 3= Sometimes, 4= Almost always, 5= Always), in which the overall score ranges from 13 to 65 points, and the higher the score, the greater the adherence. Three questions in this instrument have inverted scores: 1) In the last week, how often did you take your mask off to talk on the phone when you were away from home?; 2) In the last week, how often did you take your mask off to go to the bathroom when you were away from home?; 3) In the last week, how often did you take your mask off to talk to someone when you were away from home?. These questions were scored as follows: Never= 5 points, Rarely= 4 points, Sometimes= 3 points, Almost always= 2 points, and Always= 1 point. The instrument was applied before the start of the interventions and then on a weekly basis, after the messages had been transmitted.

### Data collection

The study protocol followed these steps: Individuals were recruited by means of an invitation sent via WhatsApp^®^ from the researchers’ contacts at all the centers, with a request to disseminate it in other groups. The invitation included information on the inclusion criteria adopted. Participants who agreed to take part in the study were first asked to confirm that they met the inclusion criteria and then electronic ally signed the Informed Consent Form. They then filled the adherence form (baseline adherence), which was made available via the Google Forms^®^ platform. The initial message was sent the next day, and subsequent messages were delivered at two-day intervals (two to three times a week) over a period of seven-week, totaling 18 messages per participant.

Data on the results was analyzed on a weekly basis, every Sunday. Participants who did not complete the full seven-week intervention or who withdrew from the study were excluded from the final analysis.

### Data analysis

The data were initially entered into Microsoft Excel^®^ version 2020, and SPSS^®^ version 25.0 statistical software was used to analyze. Initially, descriptive statistics were carried out, evaluating measures of central tendency and frequency.

The Kolmogorov-Smirnov test provided evidence that the items do not follow a normal distribution. However, since the data come from a sufficiently large sample, where, based on the central limit theorem, it was assumed that the assumption of normality did not affect the analysis of the results^(22)^. Therefore, parametric statistical tests were used to assess adherence to protective measures over the weeks. In addition, when the homogeneity of the data was confirmed, ANOVA or MANOVA tests were carried out, taking into account the sphericity of the data. A *p*-value <0.05 was considered significant.

### Ethical aspects

The study was approved by the Research Ethics Committee, decision number 4,077,371 and was registered in the Brazilian Registry of Clinical Trials (ReBEC) under number: RBR-2s94rb.

## Results

**Figure 1-:**
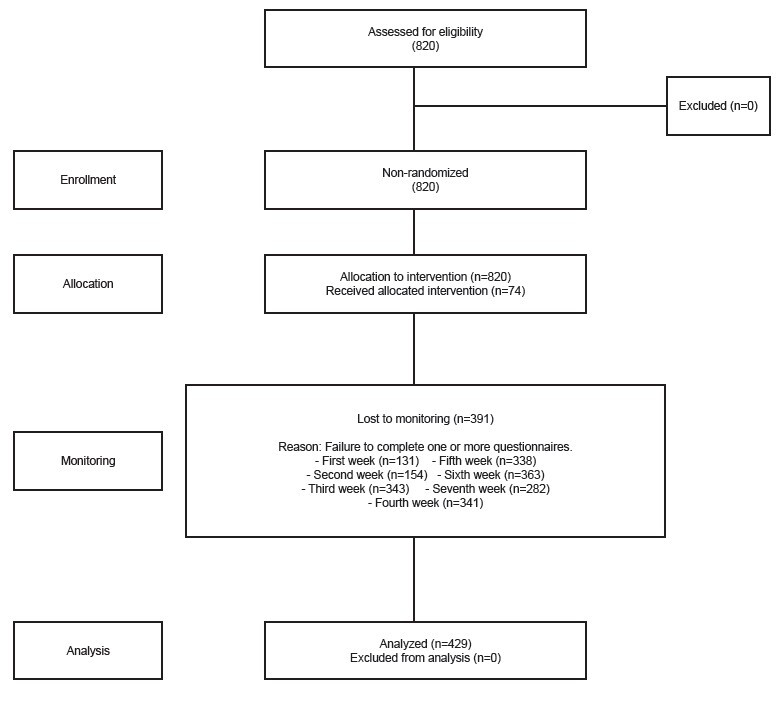
Flowchart adapted from Transparent Reporting of Evaluations with Non-randomized Designs (TREND)^(23)^

Eight hundred and twenty (820) participants were included in the study. It was necessary to increase the initially calculated sample size due to the identification, during monitoring, of a very high number of dropouts from the study, probably associated with the overload caused by the pandemic. During monitoring, 391 participants were lost, totaling 429 individuals (Figure 1).

To mitigate the impact of sample losses, a *post-hoc* analysis of statistical power was conducted based on the final sample of 429 participants. A Cohen’s effect size of 0.185, a test power of 0.80, and a significance level of 5% were observed, demonstrating that the study has sufficient power to detect the expected effects.

The participants recruited from the four Brazilian universities were from 113 different cities across 21 Brazilian states (Acre, Alagoas, Amazonas, Bahia, Ceará, Distrito Federal, Goiás, Maranhão, Mato Grosso do Sul, Minas Gerais, Pará, Paraná, Paraíba, Pernambuco, Roraima, Rio de Janeiro, Rio Grande do Norte, Rio Grande do Sul, Santa Catarina, São Paulo and Sergipe).

It was observed that the participants had a mean age of 31.02 (+10.88) years and a mean number of children of 1.82 (+0.85). Table 1 shows the other sociodemographic characteristics of the study participants.

**Table 1-:** Sociodemographic characteristics of the participants (n = 429). São Paulo, SP, Brazil, 2020

**Variable**	n* (%) ^†^
Sex: Female	327 (76.22)
Age: up 30 years	245 (57.11)
Marital Status	
Single	221 (51.51)
Married/common-law married	176 (41.03)
Divorced	28 (6.53)
Widow/widower	4 (0.93)
Family income	
Less than a minimum wage ^‡^	25 (5.83)
One to three minimum wages ^‡^	114 (26.57)
Four to seven minimum wages ^‡^	138 (32.17)
Over seven minimum wages ^‡^	152 (35.43)
Has children	163 (38.00)
Years of schooling	
Less than nine years of study	62 (14.46)
Between nine and eleven years of study	172 (40.09)
More than eleven years of study	195 (45.45)
Religion	
Catholic	157 (36.60)
Evangelical	99 (23.07)
Non-religious	94 (21.91)
Spiritist	47 (10.96)
*Umbanda* adept	16 (3.73)
Christian	7 (1.63)
Agnostic	5 (1.16)
Jehovah’s Witness	2 (0.47)
Hermeticist/Sabbatarian	2 (0.47)
Number of cohabitants	
0	51 (11.89)
1	88 (20.51)
2	104 (24.24)
3	96 (22.38)
4	62 (14.45)
More than 4 people	28 (6.53)
Employed	260 (60.61)
Formal workers	231 (88.84)
Informal workers	29 (11.16)

^*^n = Sample Number; ^†^% = Percentage; ^‡^Minimum wage = 1,039.00 Brazilian *reais* in 2020

**Figure 2-:**
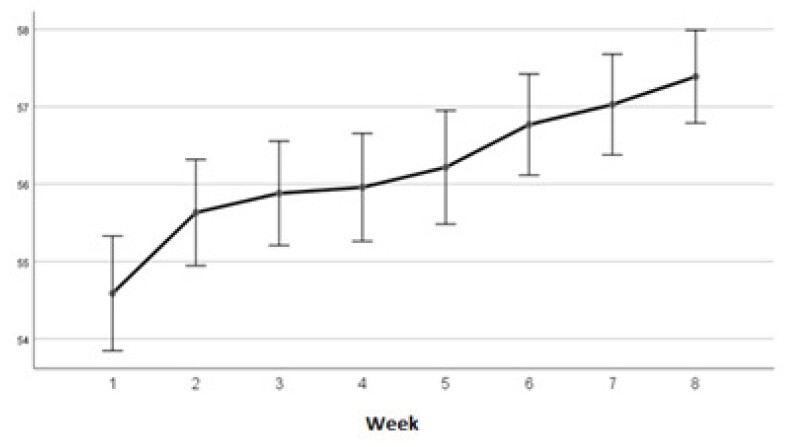
Median of the adherence score of the participants throughout the evaluations (n = 429). São Paulo, SP, Brazil, 2020

When analyzing adherence to individual protection measures against COVID-19, Figure 2 and Table 2 show that there was a progressive increase in the adherence score over the evaluations. In addition, there was a significant improvement in the last two weeks when compared to the score obtained before the intervention.

**Table 2-:** Analysis of the adherence score of the participants throughout the evaluations (n = 429). São Paulo, SP, Brazil, 2020

**Evaluation week**	**Minimum**	**Maximum**	**25%**	**Median**	**75%**	IQ*	**Mean**	**95% CI** ^†^	**SD** ^‡^	**CV** ^§^	***p* -value** ^||^	**Effect size** ^¶^	***p* -value** ^**^
Before Intervention	22.00	65.00	51.00	57.00	60.00	9.00	54.59	53.85; 55.33	7.81	14.31	<0.001	0.022	<0.001
First week	25.00	65.00	53.00	57.00	61.00	8.00	55.63	54.94; 56.32	7.24	13.01	<0.001		
Second week	25.00	65.00	52.00	57.00	61.00	9.00	55.88	55.21; 56.56	7.10	12.71	<0.001		
Third week	25.00	65.00	53.00	58.00	61.00	8.00	55.96	55.26; 56.66	7.35	13.14	<0.001		
Fourth week	23.00	65.00	53.00	58.00	61.00	8.00	56.22	55.48; 56.95	7.74	13.77	<0.001		
Fifth week	25.00	65.00	54.00	58.00	61.00	7.00	56.77	56.12; 57.42	6.89	12.13	<0.001		
Sixth week	20.00	65.00	54.00	59.00	61.00	7.00	57.03 ^††^	56.38; 57.68	6.85	12.02	<0.001		
Seventh week	28.00	65.00	55.00	59.00	62.00	7.00	57.39 ^††^	56.79; 57.99	6.32	11.02	<0.001		

*IQ = Interquartile Range; ^†^CI = Confidence Interval; ^‡^SD = Standard Deviation; ^§^CV = Coefficient of Variation; ^||^Kolmogorov-Smirnov test to assess data normality; ^¶^Partial eta squared; **MANOVA test; ^††^Means with statistically significant differences compared to baseline values after applying the Bonferroni test

Using Cronbach’s Alpha to assess reliability of the data, the instrument showed an overall alpha above 0.70, except for Question 1, which had an alpha of 0.62. Questions 06, 09, 10, 11, and 12 showed alpha values ranging from 0.74 to 0.78, while Questions 02, 03, 04, 05, 07, 08, and 13 exhibited the highest reliability, with alpha values between 0.80 and 0.90.

When analyzing each question individually and comparing them with the adherence score obtained before the start of the intervention, Table 3 shows that individual protection measure with the first improvement was the distance of one meter from people when leaving the house, showing a significant improvement at the end of the first week. The recommendations of hand hygiene when touching the front of the mask and removing the mask by the elastic band after use showed significant improvement in the third and fourth weeks, respectively. In the fifth week, significant progress was seen in measures related to changing the mask when wet, keeping the mask on when talking to someone outside the home and talking on the phone outside the home. In the sixth week, significant improvements included keeping the mask on when going to the toilet outside the home and carrying an extra mask in the bag for change. The measure of wearing the mask covering the mouth and nose showed significant improvement only in the seventh week.

Finally, four measures did not show significant changes over time: wearing a mask when leaving the house; washing the mask with soap and water when arriving home; using the mask on the subway, bus, train or in the car with another person present; and hand hygiene when touching objects outside the house.

**Table 3-:** Analysis of the adherence score of participants of each of the questions of the instrument throughout the evaluations (n = 429). São Paulo. SP, Brazil, 2020

Question	Before Intervention	First week	Second week	Third week	Fourth week	Fifth week	Sixth week	Seven week	*p* -value ^†^
	Mean (SD*)	Mean (SD*)	Mean (SD*)	Mean (SD*)	Mean (SD*)	Mean (SD*)	Mean (SD*)	Mean (SD*)	
1. In the last week, how often did you wear the mask when you left home?	4.87 (0.47)	4.86 (0.47)	4.85 (0.47) ^‡^	4.85 (0.50)	4.8 (0.64)	4.85 (0.56)	4.89 (0.46)	4.93 (0.28) ^‡^	0.004
2. In the last week, how often did you wash your mask with soap and water when you got home?	4.10 (1.34)	4.21 (1.24)	4.18 (1.26)	4.16 (1.26)	4.15 (1.30)	4.14 (1.29)	4.26 (1.20)	4.26 (1.21)	0.183
3. In the last week, how often did you take your mask off to talk on the phone when you were away from home?	4.35 (1.14)	4.42 (1.10)	4.48 (0.99)	4.48 (1.01)	4.48(1.06)	4.57 (0.93) ^§^	4.56 (0.95) ^§^	4.57(0.95) ^§^	<0.001
4. In the last week, how often did you take your mask off to go to the toilet when you were away from home?	4.38 (1.11)	4.42 (1.09)	4.48 (1.02)	4.46 (1.06)	4.49 (0.99)	4.53 (0.91)	4.56 (0.88) ^§^	4.56 (0.88) ^§^	0.004
5. In the last week, how often have you worn a mask on the subway, bus, train, or in the car in the presence of others?	3.36 (1.78)	3.48 (1.73)	3.49 (1.70)	3.39 (1.75)	3.45 (1.75)	3.45 (1.75)	3.53 (1.75)	3.49 (1.76)	0.327
6. In the last week, how often have you changed your mask whenever it was wet?	3.95 (1.43)	4.09 (1.35)	4.12 (1.31)	4.11 (1.27)	4.19 (1.25)	4.21 (1.20) ^§^	4.21 (1.22) ^§^	4.32 (1.08) ^§^	<0.001
7. In the last week, how often did you take your mask off to talk to someone when you were away from home?	4.38 (1.05)	4.43 (1.02)	4.43 (1.03)	4.43 (1.07)	4.52 (1.00)	4.55 (0.93) ^§^	4.54 (0.95) ^§^	4.59 (0.86) ^§^	<0.001
8. In the last week, how often have you used a mask that covered your mouth and nose when you were away from home?	4.71 (0.82)	4.76 (0.72)	4.76 (0.70)	4.74 (0.75)	4.76 (0.75)	4.81 (0.63)	4.83 (0.64)	4.86 (0.55) ^§^	<0.001
9. In the past week, how often did you carry an extra mask in your bag to change it if necessary?	3.90 (1.48)	4.05 (1.38)	4.07 (1.37)	4.12 (1.33)	4.13 (1.31)	4.12 (1.37)	4.15 (1.31)	4.15 (1.30)	0.024
10. In the past week, how often did you maintain a distance of one meter from others when you left home?	3.89 (1.13)	4.09 (0.96) ^§^	4.13 (0.97) ^§^	4.11 (1.03) ^§^	4.16 (1.04) ^§^	4.18 (1.0) ^§^	4.22 (0.92) ^§^	4.24 (0.93) ^§^	<0.001
11. In the past week, how often did you take your mask away by the ear loops after use?	4.35 (1.08)	4.48 (0.95)	4.50 (0.95)	4.53 (0.97)	4.54 (0.94) ^§^	4.65 (0.79) ^§^	4.65 (0.79) ^§^	4.68 (0.72) ^§^	<0.001
12. In the past week, how often did you sanitize your hands or use 70% alcohol after touching the front of your mask?	4.05 (1.14)	4.02 (1.13)	4.08 (1.14)	4.22 (1.03) ^§^	4.21 (1.07) ^§^	4.32 (0.95) ^§^	4.28 (0.98) ^§^	4.30 (0.97) ^§^	<0.001
13. In the past week, how often did you sanitize your hands or use 70% alcohol after touching an object outside your home?	4.31 (0.96)	4.32 (0.93)	4.31 (0.96)	4.36 (0.96)	4.35 (0.95)	4.40 (0.88)	4.38 (0.95)	4.43 (0.88)	0.150

*SD = Standard Deviation; ^†^MANOVA Test; ^‡^Means with statistically significant differences between the weeks following the application of the Bonferroni test; ^§^Means with statistically significant differences compared to baseline values after applying the Bonferroni test

## Discussion

This quasi-experimental study aimed to assess the effectiveness of text messages delivered via cell phones in promoting individual protective measures against COVID-19 among Brazilians from different regions. Despite the inherent limitations of this study design, such as the absence of a randomized control group, which could affect the internal validity of the results, the researchers opted for this approach due to ethical considerations. During the data collection period, Brazil was facing significant governmental discrepancies in its response to the COVID-19 pandemic, leaving the population without clear guidelines on how to proceed^(24)^.

Rhetorical analysis of risk communication during the COVID-19 pandemic in Brazil reveals significant disorganization and conflicting messages from both national and state authorities. This approach created widespread uncertainty and undermined adherence to preventive measures, such as social distancing, which were inconsistently promoted by the Presidency of the Republic and the Ministry of Health. In addition, the frequent changes in health ministers and growing political polarization have undermined the coordination of public health policies, severely affecting the country’s response to the pandemic^(25)^.

At the state level, however, risk communication was more in line with WHO guidelines, advocating measures such as horizontal social distancing. However, states also faced significant challenges, including an inefficient testing system and shortage of resource. The conflicting messages between federal and state governments created an environment of insecurity, leaving the population—especially the most vulnerable—unsure about the best ways to protect themselves^(25)^.

This uncertainty created a fertile ground for the spread of fake news and political crises. Consequently, the greater the access to information based on the best scientific evidence, the greater the adherence to COVID-19 prevention measures, and ultimately, the lower the incidence and mortality associated with the disease.

The results indicated an improvement in adherence over the course of the evaluations. Similar results have been reported in other studies that have also examined the effectiveness of text messages in various healthcare settings^(20,26-27)^.

Researchers from the United States of America (USA) conducted a pilot intervention study to evaluate the impact of text messages on medication adherence among adolescents who received liver transplantation. The results indicated that participants who received text messages, whether of praise or usual care, showed a significant increase in adherence to prescribed doses (OR: 2.49, *p*=0.03), took their medications as instructed (OR: 2.39, *p*=0.04) and demonstrated greater confidence in treatment (OR: 2.46, *p*=0.04)^(20)^.

A Brazilian study that evaluated the effectiveness of text messages and telephone counseling in smoking cessation among hospitalized smokers found a higher abstinence rate in the group that received telephone messages^(26)^.

A randomized clinical trial with 2,000 US healthcare workers, which analyzed the effect of text messages on adherence to COVID-19vaccination, showed a significant increase of 4.9 percentage points (95% CI 0.8-9.1) in adherence in the intervention group compared to the control group (*p*=0.02) after two weeks^(27)^.

The use of text messages in vulnerable populations has been shown to have significant sociocultural and economic benefits. From a socio-cultural point of view, text messages increase access to health information and improve adherence to preventive measures, while respecting local and cultural particularities. From an economic perspective, they offer a cost-effective solution for disseminating information and can help reduce healthcare costs by promoting better adherence to treatment and preventing health complications. These results highlight the potential of text messages to play a pivotal role in public health strategies, especially in contexts of vulnerability^(28)^.

In the present study, there was a significant improvement in adherence to personal protection measures at the sixth and seventh weeks compared to the score obtained before the intervention. The literature suggests that improvement in adherence generally occur between the first and third month of the intervention^(27,29-31)^, which is consistent with the results found in this study.

There is no consensus in the literature on the ideal duration of the intervention and the frequency of sending messages, which can vary from daily every three days, for periods of two weeks to six months^(12,32)^. The choice of intervention time and number of messages will depend on the outcome measured^(33-34)^. However, the studies emphasize that, regardless of the duration and frequency, the intervention should be attractive and not tedious, in order to avoid abandoning the recommendations^(34-35)^.

When analyzing each question individually, it was observed that the individual protection measure with the earliest adherence was social distancing (one meter away from other people) when leaving the house, which improved significantly at the end of the first week. It is believed that this behavior may be linked to the fact that the population perceives this measure as protective, as well as being a more direct and easier action to implement. A cross-sectional study of 2,013 adults in the US and Europe showed that motivations for social distancing were protecting everyone (86%), protecting oneself (84%) and a sense of responsibility to protect the community (84%)^(36)^.

On the other hand, four individual protection recommendations did not show significant changes over time: wearing a mask when leaving the house, washing the mask with soap and water when arriving home, wearing a mask on the subway, bus, train or car when another person was present, and sanitizing hand when touching objects outside the house. The lack of significant improvement in mask use when leaving house may be associated with the high adherence score recorded since the first evaluation, in which this measure presented the highest score. Since the beginning of the pandemic, the use of masks has been widely promoted and demanded by public agencies as one of the main measures to control the disease, resulting in greater awareness and adherence by the population^(37)^.

However, the recommendation with the lowest score since the first evaluation was the use of masks on the subway, bus, train or in the car when someone else was present. This result may have been influenced by the lack of the “not applicable” alternative for participants who traveled on foot or alone in the car, which may have led many to check the “never” option, reducing the score of this question. Therefore, this result should be interpreted with caution.

A study highlights that the COVID-19 pandemic caused global changes in transport and in the reasons and frequencies of travel, in which a significant drop in the volume of passengers in public transport was observed^(38)^. A study identified that 90% of the interviewees totally reduced (47%) or limited (44%) the use of public transport, mainly due to the change of work and/or school classes from face-to-face to remote, in addition to the fear of contracting the disease^(39)^.

The absence of a significant increase in adherence to washing masks with soap and water when arriving home can be attributed to the lack of dissemination of this measure by government agencies and the media. In addition, we can infer that these results were related to work overload during lockdown. A study that analyzed the challenges faced by remote work professionals during lockdown identified family-related difficulties, such as increased responsibilities for school, childcare, and household chores; work-related problems, such as long hours and inadequate space^(40)^; and mental health problems, such as symptoms of anxiety and depression, exhaustion, and burnout^(40-41)^.

The low adherence to hand hygiene when touching objects outside the home can be explained by the lack of access or adequate material to carry out this protection measure. A study conducted in the city of Woldia, northeastern Ethiopia, observed that the majority of participants (63.1%, n= 255/404) did not practice hand hygiene in all locations due to restrictions on access to water and hygiene products^(42)^.

Given the results obtained, text messages sent by cell phone demonstrated to be an effective tool for education in various health contexts, including the prevention of emerging diseases. This approach allows reaching broad groups of people, contributing to the transmission of relevant and timely information and, consequently, to the improvement of adherence to treatment and protective measures.

The findings presented can serve as a guide for health managers in developing prevention programs and public policies for emergency health situations similar to the COVID-19 pandemic. In addition, these results will empower healthcare professionals, particularly nurses, to design targeted health education strategies for groups more susceptible to non-adherence, thereby enhancing the effectiveness of interventions and fostering greater compliance with protective measures.

This study has some limitations. It was not possible to verify whether all participants had read the messages. In addition, as it was an emerging disease, there was no validated instrument to assess adherence to individual protection recommendations. Considering the urgency of interventions to improve the population’s knowledge and adherence, the instrument used was developed by the researchers at the main center based on the recommendations available at the time and only refined in collaboration with researchers from the other centers. Furthermore, the instrument was not tested with the target population before the study began, which may have affected some of the results found and discussed above. The question about the use of masks in public transport did not have an alternative “not applicable”, which may have impacted the results.

Confounding variables, such as age, educational level, income, and changes in public policies, were not controlled in this study. The lack of control over these variables limits the ability to establish direct causal relationships between the intervention and the changes observed. Finally, the observed loss of samples may have affected the results, limiting the generalizability of the findings. Therefore, the effects identified should be interpreted with caution. Despite the aforementioned limitations, the results are relevant and provide important insights for public policies and health communication strategies.

## Conclusion

Messages sent by cell phone were effective in promoting adherence to COVID-19 prevention practices, especially after the sixth week of sending. This educational tool may be considered for future pandemics, due to its low cost and proven effectiveness in improving knowledge and adherence in various contexts.

The findings presented can serve as a guide for health managers in the development of prevention programs and public policies for emergency health similar to the COVID-19 pandemic. In addition, researchers may use this protocol to guide intervention in future studies. However, due to the significant number of losses during the follow-up period, it is recommended to reduce the duration of follow-up to four weeks.

Furthermore, it is recommended that future studies assess the validity evidence of the instrument developed and use a randomized design to further strengthen the results.

## Data Availability

All data generated or analysed during this study are included in this published article.
