# Supplementary material for: Effectiveness of text messages in the adherence to COVID-19 prevention practices: a quasi-experimental study
Source: Rev Lat Am Enfermagem. 2025 Dec 1;33:e4707. doi: 10.1590/1518-8345.7810.4707 (PMC12685359; doi:10.1590/1518-8345.7810.4707)
Supplement: Translation

# Translation: Eficácia de mensagens de texto na adesão às práticas de prevenção de COVID-19: um estudo quasi-experimental*

**DOI:** 10.1590/1518-8345.7810.4708

**Authors:** Vinicius Lino de Souza, Sheila Coelho Ramalho Vasconcelos Morais, Rafaela Batista dos Santos Pedrosa, Mirian Ueda Yamaguchi, Eduesley Santana Santos, Agueda Maria Ruiz Zimmer Cavalcante, Vinícius Batista Santos, Juliana de Lima Lopes

**Affiliations:** 1 Universidade Federal de São Paulo, Escola Paulista de Enfermagem, São Paulo, SP, Brasil. Universidade Federal de São Paulo Escola Paulista de Enfermagem SP São Paulo Brasil; 2 Bolsista do Conselho Nacional de Desenvolvimento Científico e Tecnológico (CNPq), Brasil. Bolsista do Conselho Nacional de Desenvolvimento Científico e Tecnológico Brasil; 3 Universidade Federal do Pernambuco, Departamento de Enfermagem, Recife, PE, Brasil. Universidade Federal do Pernambuco Departamento de Enfermagem PE Recife Brasil; 4 Universidade Estadual de Campinas, Departamento de Enfermagem, Campinas, SP, Brasil. Universidade Estadual de Campinas Departamento de Enfermagem SP Campinas Brasil; 5 Centro Universitário de Maringá, Departamento de Saúde, Maringá, PR, Brasil. Centro Universitário de Maringá Departamento de Saúde PR Maringá Brasil; 6 Universidade Federal de Sergipe, Departamento de Enfermagem, Maceió, SE, Brasil. Universidade Federal de Sergipe Departamento de Enfermagem SE Maceió Brasil; 7 Universidade Federal do Goiás, Faculdade de Enfermagem, Goiânia, GO, Brasil. Universidade Federal do Goiás Faculdade de Enfermagem GO Goiânia Brasil

**Keywords:** Envio de Mensagens de Texto, Coronavírus, Isolamento Social, Máscaras, Cooperação e Adesão ao Tratamento, Terapia Comportamental

## Abstract

analisar a eficácia das mensagens enviadas por celular na adesão ao uso de máscaras, higienização das mãos e distanciamento social durante a pandemia de COVID-19.

estudo multicêntrico do tipo ensaio clínico quasi-experimental, realizado em quatro universidades brasileiras entre julho e outubro de 2020. A amostra consistiu em 429 pessoas de diferentes cidades brasileiras que receberam mensagens a cada dois dias durante sete semanas sobre medidas de proteção individual. A adesão às medidas foi analisada por um instrumento desenvolvido pelos pesquisadores do centro principal e revisado pelos demais pesquisadores, composto por 13 perguntas e uma escala Likert de cinco pontos, com a pontuação global variando de 13 a 65 pontos, em que quanto maior a pontuação, maior a adesão. A análise estatística foi realizada por meio de análise multivariada de variância (MANOVA), considerando*p*< 0,05 como significativo.

foi observada uma melhora na pontuação de adesão ao longo das semanas. Quando comparada com a pontuação obtida antes da intervenção, houve uma melhora significativa na adesão nas duas últimas semanas (*p*< 0,001).

a intervenção por meio de mensagens enviadas por telefone celular se mostrou eficaz na promoção da adesão às práticas de proteção individual contra a COVID-19. No entanto, a perda de amostra observada pode ter afetado os resultados, limitando sua generalização. Portanto, os efeitos identificados devem ser interpretados com cautela. Registro Brasileiro de Ensaios Clínicos (RBR-2s94rb).

## Destaques

**(1)** Uso de tecnologia de baixo custo para promover a educação em saúde. **(2)** Estratégia para fortalecer ou melhorar políticas de saúde. **(3)** O protagonismo da enfermagem como articuladora da estratégia de adesão.

## Introdução

A COVID-19, causada pelo vírus SARS-CoV-2, foi identificada como um problema de saúde global e declarada uma pandemia pela Organização Mundial da Saúde (OMS) em 11 de março de 2020^(1)^. Embora a COVID-19 não seja mais classificada como uma pandemia, ainda são observados casos de hospitalização e mortes relacionadas à doença^(2-3)^.

No início da pandemia, governos de diversos países adotaram uma série de estratégias para conter a disseminação do SARS-CoV-2 e mitigar seus impactos na saúde e na economia, como testes comunitários, triagem de contatos, isolamento e medidas de proteção individual^(4)^.

As recomendações para proteção pessoal estavam relacionadas ao uso correto e à remoção de máscaras faciais, distância social de um metro entre as pessoas e higienização das mãos com água e sabão ou álcool 70%^(5-6)^. Estas medidas não eram práticas comuns realizadas pela população brasileira, o que configurava um desafio ainda maior para os governantes do país. Pesquisadores apontaram que, devido à alta transmissibilidade do vírus e à ausência de tratamento medicamentoso e vacinação contra a COVID-19 no início da pandemia, o controle da doença só seria possível por meio destas mudanças comportamentais de forma massiva e rápida^(7)^.

Outro fator que se tornou um desafio no início da pandemia foi a escassez de máscaras cirúrgicas, em que a OMS recomendou que estas máscaras fossem reservadas aos profissionais de saúde, às pessoas com sintomas da doença e aos cuidadores ou àqueles que compartilhavam o mesmo espaço com pessoas com suspeita de COVID-19 ou com sintomas respiratórios^(5)^.

A OMS também afirmou que os tomadores de decisão em cada país deveriam aconselhar sobre o uso de máscaras não cirúrgicas em relação ao número de camadas e tipos de tecido e ao formato e ajuste da máscara^(5)^. Em vista destas recomendações, a Agência Nacional de Vigilância Sanitária (ANVISA) recomendou que as máscaras não cirúrgicas fossem compostas por três camadas de tecido, sendo a camada externa de tecido não impermeável, a central de tecido respirável e a camada interna de tecido de algodão^(6)^.

Apesar da necessidade de tantas mudanças na rotina diária da população, alguns estudos identificaram que a população pretendia praticar medidas de proteção individual contra a COVID-19 e que o cumprimento de tais recomendações era maior quando associado a um maior conhecimento^(8-9)^.

Um estudo transversal realizado na Arábia Saudita com 502 participantes adultos idosos, com 65 anos ou mais, mostrou que mais da metade dos participantes (52,2%) pretendia praticar medidas de proteção individual contra a COVID-19. Além disso, as chances de praticar as recomendações preventivas eram 1,59 vezes (IC 95% = 1,01-2,52) nas mulheres em comparação com os homens e 2,72 vezes (IC 95% = 1,44-5,16) maiores em indivíduos com alto nível de escolaridade^(8)^.

Resultados semelhantes foram encontrados nos Estados Unidos da América (EUA), onde uma pesquisa com 506 residentes revelou que os participantes que perceberam as práticas de saúde recomendadas pelo Centro de Controle e Prevenção de Doenças dos Estados Unidos (CDC) como eficazes tinham intenções mais fortes de se envolver nestas práticas. Além disso, a percepção dos participantes sobre a gravidade de COVID-19 nos EUA foi um fator significativo (β = 0,48; *p* ≤ 0,001; 95% IC = 0,418–0,539). A eficácia percebida das práticas recomendadas foi responsável pela maior variação nas intenções comportamentais (β = 0,19; *p*≤0,001; 95% IC=0,112–0,260)^(9)^.

Neste sentido, as intervenções de educação em saúde para melhorar o conhecimento da população e a adesão às medidas de proteção individual tornaram-se essenciais e fundamentais para o controle da doença. As ações de educação em saúde são uma prática cotidiana realizada pelos profissionais de enfermagem e várias estratégias podem ser adotadas, como o envio de mensagens^(10-11)^.

Uma metanálise que avaliou nove ensaios clínicos randomizados com 1.121 participantes mostrou um impacto favorável do envio de mensagens por telefone celular quando comparado aos cuidados habituais na adesão à medicação entre indivíduos com diabetes mellitus tipo 2 (diferença média padronizada: 0,36; 95% IC: 0,14-0,59)^(12)^.

Um ensaio clínico randomizado avaliou se os estímulos comportamentais fornecidos por meio de mensagens de texto poderiam acelerar a adesão à política de vacinação contra a COVID-19 de um sistema de saúde entre 2.000 funcionários de sistemas de saúde no Centro-Oeste ou no Sul dos EUA que não estavam aderindo à política de vacinação, um mês antes do prazo final. Os resultados revelaram que, ao final da intervenção de duas semanas, 363 participantes do grupo de incentivo por mensagem de texto (36,3%) e 318 participantes do grupo controle (31,8%) haviam aderido à política de vacinação, representando um aumento significativo de 4,9 pontos percentuais (IC 95%, 0,8 a 9,1; *p*=0,002) em análises ajustadas, comparando o grupo de incentivo com o grupo controle. Entre os participantes que aderiram até o final do período de acompanhamento de quatro semanas, o estímulo por mensagem de texto reduziu significativamente o tempo de adesão em uma média de 2,4 dias (95% IC, 2,1 a 4,7; *p*<0,001) e uma mediana de 5,0 dias (95% IC, 2,5 a 7,7;*p*<0,001), comparado ao grupo controle^(13)^.

Estes resultados estão de acordo com outra metanálise que analisou o efeito das mensagens para o controle do excesso de peso e incluiu 12 estudos controlados e randomizados. Dez dos estudos incluídos mostraram um efeito significativo na perda de peso dos participantes e a diferença média agrupada na mudança do índice de massa corporal após a intervenção foi de -0,43 kg/m² (95% IC -0,63 a -0,23 kg/m²)^(14)^.

No Brasil, as mensagens de texto são um meio de comunicação amplamente utilizado, especialmente em regiões onde o acesso à Internet é limitado ou onde o uso de smartphones avançados ainda não é generalizado^(15)^. Este recurso é especialmente valioso em contextos de saúde pública, pois facilita a rápida disseminação de informação e atinge diversos segmentos da população, incluindo aqueles com níveis mais baixos de educação ou acesso limitado a outras fontes de informação^(15)^. Além disso, fatores culturais, como a confiança na comunicação direta, podem afetar a forma como as mensagens são recebidas e o grau de adesão às orientações^(16)^.

Embora existam pesquisas sobre campanhas de saúde pública e estratégias de persuasão, as evidências sobre como estas mensagens influenciam especificamente as populações durante crises de saúde em larga escala, como a pandemia de COVID-19, permanecem limitadas. Além disso, embora várias abordagens persuasivas, incluindo mensagens pró-sociais, prova social e apelos morais estejam documentadas na literatura, poucos estudos exploraram o impacto destas estratégias no contexto de doenças infecciosas emergentes, especialmente considerando a politização que marcou a pandemia de COVID-19. Portanto, é essencial investigar como esta estratégia afetou a adesão a comportamentos preventivos em um contexto tão singular.

Assim sendo, o objetivo deste estudo foi analisar a eficácia das mensagens enviadas por telefone celular na adesão ao uso de máscaras, higienização das mãos e distanciamento social durante a pandemia de COVID-19.

## Método

### Design

Este é um estudo clínico quasi-experimental e multicêntrico conduzido de acordo com as diretrizes do *Transparent Reporting of Evaluations with Non-randomized Designs* (TREND).

### Local

Este estudo foi realizado em quatro universidades públicas dos estados brasileiros de São Paulo, Paraná, Pernambuco e Sergipe.

### Período

A coleta de dados foi realizada de julho a outubro de 2020.

### População e critérios de seleção

A população do estudo foi composta por adultos brasileiros. Os critérios de elegibilidade incluíram: residir no Brasil, ter 18 anos de idade ou mais, ter um telefone celular e acesso à Internet para usar o aplicativo *WhatsApp*®, não ter deficiências visuais e/ou cognitivas, ser alfabetizado e ser proficiente em português.

### Definição da amostra

O tamanho da amostra foi calculado usando o *software G Power*®, versão 3.1.9.2 (disponível em https://www.gpower.hhu.de/). Com base em um tamanho de efeito de Cohen de 0,15, um poder de teste de 0,80 e um nível de significância de 5%, a amostra mínima necessária foi determinada em 351 participantes. Para compensar possíveis perdas durante o monitoramento, foi adicionado 20% ao tamanho da amostra, resultando em um tamanho mínimo de 421 participantes.

### Intervenções

A intervenção consistiu no envio de mensagens previamente validadas^(17)^. O conteúdo das mensagens de texto foi desenvolvido por meio de uma revisão narrativa da literatura, com o objetivo de analisar as recomendações relacionadas ao uso de máscara, higiene das mãos e distanciamento social durante a pandemia de SARS-CoV-2.

Foram criadas 18 mensagens, abrangendo tópicos como a doença, os métodos de transmissão e as medidas preventivas relacionadas ao distanciamento social, ao uso de máscara e à higiene das mãos. Estas mensagens foram elaboradas usando frases concisas e um tom orientado à ação para promover a adoção de medidas preventivas contra a COVID-19, acompanhadas de imagens ilustrativas relevantes para cada tópico^(17)^. Veja a seguir um exemplo do texto de uma das mensagens desenvolvidas: “*A máscara é uma medida de proteção contra o coronavírus, e você deve sempre usá-la quando sair de casa*”^(17)^.

Após o desenvolvimento, as mensagens foram avaliadas por um grupo de especialistas, que as examinou com base na relevância teórica, clareza, aplicabilidade prática e vocabulário. A validade do conteúdo foi verificada por meio do cálculo do Índice de Validade do Conteúdo (IVC), com duas rodadas de avaliação, necessárias para que as mensagens atingissem um IVC superior a 90%, garantindo sua validade^(17)^.

Estas mensagens foram enviadas por telefone celular de duas a três vezes por semana, com um intervalo de dois dias entre cada envio, durante sete semanas. A decisão de usar um intervalo de dois dias foi informada por um estudo anterior que examinou o impacto do envio de mensagens de texto com esta frequência, com foco na adesão e no autocuidado em indivíduos com doença arterial coronariana. Neste estudo, a maioria dos participantes relatou estar satisfeita com o recebimento de mensagens com um intervalo de dois dias entre cada envio^(18)^. A literatura não oferece consenso sobre a frequência ideal para o envio de mensagens, com intervalos que variam de mensagens diárias a semanais ou quinzenais^(12,19)^. O período de sete semanas para a intervenção foi adotado com base em estudos que utilizaram esta estratégia de educação em saúde com outras populações e que empregaram períodos de monitoramento semelhantes^(20-21)^.

### Variáveis e instrumentos

A adesão foi avaliada por meio de um instrumento desenvolvido inicialmente por pesquisadores do principal centro de estudos e posteriormente refinado por oito profissionais de saúde com mestrado ou doutorado em doenças transmissíveis ou infecciosas. Cada especialista tinha pelo menos três anos de experiência em doenças infecciosas ou no gerenciamento de situações relacionadas a pandemias. O processo de refinamento foi conduzido por meio de uma reunião virtual moderada pelo supervisor do estudo. Durante a sessão, cada item do instrumento foi cuidadosamente revisado, com discussões contínuas até que os especialistas chegassem a um consenso.

Este instrumento, que está de acordo com as mensagens usadas na intervenção, contém 13 perguntas sobre o uso correto de máscaras faciais, a importância da higiene das mãos e o distanciamento social. Cada item do instrumento tem uma escala Likert de cinco pontos (1= Nunca, 2= Raramente, 3= Às vezes, 4= Quase sempre, 5= Sempre), no qual a pontuação geral varia de 13 a 65 pontos, e quanto maior a pontuação, maior a adesão. Três perguntas deste instrumento têm pontuações invertidas: 1) Na última semana, com que frequência você retirou a máscara para falar ao telefone quando estava fora de casa?; 2) Na última semana, com que frequência você retirou a máscara para ir ao banheiro quando estava fora de casa?; 3) Na última semana, com que frequência você retirou a máscara para falar com alguém quando estava fora de casa? Estas perguntas foram pontuadas da seguinte forma: Nunca = 5 pontos, Raramente = 4 pontos, Às vezes = 3 pontos, Quase sempre = 2 pontos e Sempre = 1 ponto. O instrumento foi aplicado antes do início das intervenções e, depois, semanalmente, após o início da transmissão das mensagens.

### Coleta de dados

O protocolo do estudo seguiu as seguintes etapas: Os indivíduos foram recrutados por meio de convite enviado pelo *WhatsApp*® a partir dos contatos dos pesquisadores de todos os centros, com solicitação de divulgação em outros grupos. O convite incluía informação sobre os critérios de inclusão adotados.

Os participantes que concordaram em participar do estudo foram primeiramente solicitados a confirmar se atendiam aos critérios de inclusão e, em seguida, assinaram eletronicamente o Termo de Consentimento Livre e Esclarecido. Em seguida, eles preencheram o formulário de adesão (adesão à linha de base), que foi disponibilizado por meio da plataforma *Google Forms*®. A mensagem inicial foi enviada no dia seguinte, e as mensagens subsequentes foram entregues em intervalos de dois dias (duas a três vezes por semana) em um período de sete semanas, totalizando 18 mensagens por participante.

Os dados foram analisados semanalmente, todos os domingos. Os participantes que não concluíram a intervenção completa de sete semanas ou que se retiraram do estudo foram excluídos da análise final.

### Análise dos dados

Os dados foram inicialmente inseridos no *Microsoft Excel*^®^ versão 2020 e para a análise estatística dos dados foi utilizado o *software* estatístico SPSS^®^ versão 25.0. Inicialmente, foi realizada a estatística descritiva, com avaliação das medidas de tendência central e frequência.

O teste de Kolmogorov-Smirnov forneceu evidências de que os itens do instrumento não seguem uma distribuição normal. No entanto, como os dados são provenientes de uma amostra suficientemente grande, com base no teorema do limite central, presumiu-se que a suposição de normalidade não afeta a análise dos resultados^(22)^. Portanto, foram usados testes estatísticos paramétricos para avaliar a adesão às medidas de proteção ao longo das semanas. Além disso, quando a homogeneidade dos dados foi confirmada, foram realizados testes ANOVA ou MANOVA, levando em conta a esfericidade dos dados. Um valor de *p*<0,05 foi considerado significativo.

### Aspectos éticos

O estudo foi aprovado pelo Comitê de Ética em Pesquisa, parecer número 4.077.371, e foi registrado no Registro Brasileiro de Ensaios Clínicos (ReBEC), númeroRBR-2s94rb.

## Resultados

Oitocentos e vinte (820) participantes foram incluídos no estudo. Houve a necessidade de aumentar o tamanho da amostra calculada devido à identificação, ao longo do monitoramento, de uma quantidade muito alta de desistências do estudo, provavelmente associadas à sobrecarga causada pela pandemia. Durante o monitoramento, houve uma perda de 391 participantes, totalizando 429 indivíduos analisados (Figura 1).

**Figura 1-:** Fluxograma adaptado de Transparent Reporting of Evaluations with Non-randomized Designs (TREND)^(23)^

Para atenuar o impacto das perdas de amostra, foi realizada uma análise post-hoc do poder estatístico com base na amostra final de 429 participantes. Foi observado um tamanho de efeito de Cohen de 0,185, um poder de teste de 0,80 e um nível de significância de 5%, demonstrando que o estudo tem poder suficiente para detectar os efeitos esperados.

Os participantes recrutados das quatro universidades brasileiras eram de 113 cidades diferentes em 21 estados brasileiros (Acre, Alagoas, Amazonas, Bahia, Ceará, Distrito Federal, Goiás, Maranhão, Mato Grosso do Sul, Minas Gerais, Pará, Paraná, Paraíba, Pernambuco, Roraima, Rio de Janeiro, Rio Grande do Norte, Rio Grande do Sul, Santa Catarina, São Paulo e Sergipe).

Observou-se que os participantes tinham uma média de idade de 31,02±10,88 anos e uma média de número de filhos de 1,82±0,85. A Tabela 1 mostra as outras características sociodemográficas dos participantes do estudo.

**Tabela 1-:** Características sociodemográficas dos participantes (n = 429). São Paulo, SP, Brasil, 2020

**Variável**	n* (%) ^†^
Sexo: Feminino	327 (76,22)
Idade: até 30 anos	245 (57,11)
Estado civil	
Solteiro (a)	221 (51,51)
Casado (a)/União estável	176 (41,03)
Divorciado (a)	28 (6,53)
Viúvo (a)	4 (0,93)
Renda familiar	
Menos de um salário-mínimo ^‡^	25 (5,83)
De um a três salários-mínimos ^‡^	114 (26,57)
De quatro a sete salários-mínimos ^‡^	138 (32,17)
Mais de sete salários-mínimos ^‡^	152 (35,43)
Têm filhos	163 (38,00)
Anos de escolaridade	
Menos de nove anos de estudo	62 (14,46)
Entre nove e onze anos de estudo	172 (40,09)
Mais de onze anos de estudo	195 (45,45)
Religião	
Católica	157 (36,60)
Evangélica	99 (23,07)
Não religioso (a)	94 (21,91)
Espírita	47 (10,96)
Adepto da Umbanda	16 (3,73)
Cristão	7 (1,63)
Agnóstico	5 (1,16)
Testemunha de Jeová	2 (0,47)
Hermetista/Sabatista	2 (0,47)
Número de coabitantes	
0	51 (11,89)
1	88 (20,51)
2	104 (24,24)
3	96 (22,38)
4	62 (14,45)
Mais de 4 pessoas	28 (6,53)
Empregado	260 (60,61)
Empregado registrado	231 (88,84)
Empregado não registrado	29 (11,16)

*n = Número de pessoas; ^†^% = Porcentagem; ^‡^Salário-mínimo = R$ 1.039,00 em 2020

Ao analisar a adesão às medidas de proteção individual contra a COVID-19, observa-se na Figura 2 e na Tabela 2 que houve um aumento progressivo da pontuação da adesão ao longo das avaliações. Além disso, verificou-se que os indivíduos tiveram uma melhora significativa nas duas últimas semanas quando comparados com a pontuação obtida antes da intervenção.

**Figura 2-:** Mediana da pontuação da adesão dos participantes durante as avaliações (n = 429). São Paulo, SP, Brasil, 2020

**Tabela 2-:** Análise da pontuação de adesão dos participantes durante as avaliações (n = 429). São Paulo, SP, Brasil, 2020

**Semana de avaliação**	**Mínimo**	**Máximo**	**25%**	**Mediana**	**75%**	QI*	**Média**	**95% IC** ^†^	**DP** ^‡^	**CV** ^§^	**Valor de *p*** ^||^	**Tamanho do efeito** ^¶^	**Valor de *p*** ^**^
Antes da intervenção	22,00	65,00	51,00	57,00	60,00	9,00	54,59	53,85; 55,33	7,81	14,31	<0,001	0,022	<0,001
Primeira semana	25,00	65,00	53,00	57,00	61,00	8,00	55,63	54,94; 56,32	7,24	13,01	<0,001		
Segunda semana	25,00	65,00	52,00	57,00	61,00	9,00	55,88	55,21; 56,56	7,10	12,71	<0,001		
Terceira semana	25,00	65,00	53,00	58,00	61,00	8,00	55,96	55,26; 56,66	7,35	13,14	<0,001		
Quarta semana	23,00	65,00	53,00	58,00	61,00	8,00	56,22	55,48; 56,95	7,74	13,77	<0,001		
Quinta semana	25,00	65,00	54,00	58,00	61,00	7,00	56,77	56,12; 57,42	6,89	12,13	<0,001		
Sexta semana	20,00	65,00	54,00	59,00	61,00	7,00	57,03 ^††^	56,38; 57,68	6,85	12,02	<0,001		
Sétima semana	28,00	65,00	55,00	59,00	62,00	7,00	57,39 ^††^	56,79; 57,99	6,32	11,02	<0,001		

*QI = Intervalo interquartil; ^†^IC = Intervalo de Confiança; ^‡^DP = Desvio-Padrão; ^§^CV = Coeficiente de Variação; ^||^Teste Kolmogorov-Smirnov para medir normalidade dos dados; ^¶^Eta quadrado parcial; **Teste de MANOVA; ^††^Médias com diferenças estatisticamente significativas em comparação com os valores de linha de base após a aplicação do teste de Bonferroni

Usando o alfa de Cronbach para avaliar a consistência interna demonstrou-se um alfa geral acima de 0,70, com exceção da Questão 1, que teve um alfa de 0,62. As questões 06, 09, 10, 11 e 12 apresentaram valores de alfa que variaram de 0,74 a 0,78, enquanto as questões 02, 03, 04, 05, 07, 08 e 13 apresentaram a maior consistência interna, com valores de alfa entre 0,80 e 0,90.

Ao analisar cada pergunta individualmente e compará-las com o escore de adesão obtido antes do início da intervenção, a Tabela 3 mostra que a medida de proteção individual com a primeira melhora foi a distância de um metro das pessoas ao sair de casa, mostrando uma melhora significativa no final da primeira semana. As recomendações de higienização das mãos ao tocar a parte frontal da máscara e a remoção da máscara pelo elástico após o uso apresentaram melhora significativa na terceira e quarta semanas, respectivamente.

Na quinta semana, foi observado um progresso significativo nas medidas relacionadas à troca da máscara quando molhada, manutenção da máscara ao falar com alguém fora de casa e falar ao telefone fora de casa. Na sexta semana, as melhorias significativas incluíram manter a máscara ao ir ao banheiro fora de casa e carregar uma máscara extra na bolsa, para troca. A medida de usar a máscara cobrindo a boca e o nariz apresentou melhora significativa somente na sétima semana.

Por fim, quatro medidas não apresentaram mudanças significativas ao longo do tempo: usar a máscara ao sair de casa; lavar a máscara com água e sabão ao chegar em casa; usar a máscara no metrô, ônibus, trem ou no carro com outra pessoa presente; e higienizar as mãos ao tocar em objetos fora de casa.

**Tabela 3-:** Análise da pontuação da aderência dos participantes para cada uma das questões do instrumento ao longo das avaliações (n = 429). São Paulo, SP, Brasil, 2020

Questões	Antes da intervenção	Primeira semana	Segunda semana	Terceira semana	Quarta semana	Quinta semana	Sexta semana	Sétima semana	Valor de *p* ^†^
	Média (DP*)	Média (DP*)	Média (DP*)	Média (DP*)	Média (DP*)	Média (DP*)	Média (DP*)	Média (DP*)	
1. Na última semana, com que frequência você usou a máscara quando saiu de casa?	4,87 (0,47)	4,86 (0,47)	4,85 (0,47) ^‡^	4,85 (0,50)	4,8 (0,64)	4,85 (0,56)	4,89 (0,46)	4,93 (0,28) ^‡^	0,004
2. Na última semana, com que frequência você lavou a máscara com água e sabão quando chegou em casa?	4,10 (1,34)	4,21 (1,24)	4,18 (1,26)	4,16 (1,26)	4,15 (1,30)	4,14 (1,29)	4,26 (1,20)	4,26 (1,21)	0,183
3. Na última semana, com que frequência você retirou a máscara para falar ao telefone quando estava fora de casa?	4,35 (1,14)	4,42 (1,10)	4,48 (0,99)	4,48 (1,01)	4,48 (1,06)	4,57 (0,93) ^§^	4,56 (0,95) ^§^	4,57 (0,95) ^§^	<0,001
4. Na última semana, com que frequência você retirou a máscara para ir ao banheiro quando estava fora de casa?	4,38 (1,11)	4,42 (1,09)	4,48 (1,02)	4,46 (1,06)	4,49 (0,99)	4,53 (0,91)	4,56 (0,88) ^§^	4,56 (0,88) ^§^	0,004
5. Na última semana, com que frequência você usou uma máscara no metrô, ônibus, trem ou no carro na presença de outras pessoas?	3,36 (1,78)	3,48 (1,73)	3,49 (1,70)	3,39 (1,75)	3,45 (1,75)	3,45 (1,75)	3,53 (1,75)	3,49 (1,76)	0,327
6. Na última semana, com que frequência você trocou sua máscara quando ela estava molhada?	3,95 (1,43)	4,09 (1,35)	4,12 (1,31)	4,11 (1,27)	4,19 (1,25)	4,21 (1,20) ^§^	4,21 (1,22) ^§^	4,32 (1,08) ^§^	<0,001
7. Na última semana, com que frequência você retirou a máscara para falar com alguém quando estava fora de casa?	4,38 (1,05)	4,43 (1,02)	4,43 (1,03)	4,43 (1,07)	4,52 (1,00)	4,55 (0,93) ^§^	4,54 (0,95) ^§^	4,59 (0,86) ^§^	<0,001
8. Na última semana, com que frequência você usou uma máscara que cobria a boca e o nariz quando estava fora de casa?	4,71 (0,82)	4,76 (0,72)	4,76 (0,70)	4,74 (0,75)	4,76 (0,75)	4,81 (0,63)	4,83 (0,64)	4,86 (0,55) ^§^	<0,001
9. Na última semana, com que frequência você carregou uma máscara extra na bolsa para trocá-la, se necessário?	3,90 (1,48)	4,05 (1,38)	4,07 (1,37)	4,12 (1,33)	4,13 (1,31)	4,12 (1,37)	4,15 (1,31)	4,15 (1,30)	0,024
10. Na semana passada, com que frequência você manteve uma distância de um metro de outras pessoas quando saiu de casa?	3,89 (1,13)	4,09 (0,96) ^§^	4,13 (0,97) ^§^	4,11 (1,03) ^§^	4,16 (1,04) ^§^	4,18 (1,0) ^§^	4,22 (0,92) ^§^	4,24 (0,93) ^§^	<0,001
11. Na semana passada, com que frequência você removeu a máscara pelas alças auriculares após o uso?	4,35 (1,08)	4,48 (0,95)	4,50 (0,95)	4,53 (0,97)	4,54 (0,94) ^§^	4,65 (0,79) ^§^	4,65 (0,79) ^§^	4,68 (0,72) ^§^	<0,001
12. Na semana passada, com que frequência você higienizou as mãos ou usou álcool 70% depois de tocar a parte frontal da máscara?	4,05 (1,14)	4,02 (1,13)	4,08 (1,14)	4,22 (1,03) ^§^	4,21 (1,07) ^§^	4,32 (0,95) ^§^	4,28 (0,98) ^§^	4,30 (0,97) ^§^	<0,001
13. Na semana passada, com que frequência você higienizou as mãos ou usou álcool 70% depois de tocar em um objeto fora de casa?	4,31 (0,96)	4,32 (0,93)	4,31 (0,96)	4,36 (0,96)	4,35 (0,95)	4,40 (0,88)	4,38 (0,95)	4,43 (0,88)	0,150

*DP = Desvio-Padrão; ^†^Teste MANOVA; ^‡^Médias com diferenças estatisticamente significativas entre as semanas após a aplicação do teste de Bonferroni; ^§^Médias com diferenças estatisticamente significativas em comparação com os valores de linha de base após a aplicação do teste de Bonferroni

## Discussão

Este estudo quasi-experimental teve por objetivo avaliar a eficácia das mensagens de texto enviadas por meio de telefones celulares na promoção de medidas de proteção individual contra a COVID-19 entre brasileiros de diferentes regiões. Apesar das limitações inerentes a este desenho de estudo, como a ausência de um grupo controle randomizado, que poderia afetar a validade interna dos resultados, os pesquisadores optaram por esta abordagem em razão de considerações éticas. Durante o período de coleta de dados, o Brasil estava enfrentando discrepâncias governamentais significativas na resposta à pandemia de COVID-19, deixando a população sem diretrizes claras sobre como proceder^(24)^.

A análise retórica da comunicação de risco durante a pandemia de COVID-19 no Brasil revela uma desorganização significativa e mensagens conflitantes das autoridades nacionais e estaduais. Esta abordagem criou uma incerteza generalizada e prejudicou a adesão às medidas preventivas, como o distanciamento social, que foram promovidas de forma inconsistente pela Presidência da República e pelo Ministério da Saúde. Além disso, as frequentes mudanças de ministros da saúde e a crescente polarização política prejudicaram a coordenação das políticas de saúde pública, afetando gravemente a resposta do país à pandemia^(25)^.

Em nível estadual, no entanto, a comunicação de risco estava mais alinhada com as diretrizes da OMS, defendendo medidas como o distanciamento social horizontal. No entanto, os estados também enfrentaram desafios significativos, incluindo um sistema de testes ineficiente e a escassez de recursos. As mensagens conflitantes entre os governos federal e estadual criaram um ambiente de insegurança, deixando a população, especialmente a mais vulnerável, insegura sobre as melhores maneiras de se proteger^(25)^.

Esta incerteza criou um terreno fértil para a disseminação de notícias falsas e crises políticas. Consequentemente, quanto maior o acesso à informação baseada nas melhores evidências científicas, maior a adesão às medidas de prevenção da COVID-19 e, por fim, menor a incidência e mortalidade associadas à doença.

Os resultados indicaram uma melhora na adesão ao longo das avaliações. Resultados semelhantes foram relatados em outros estudos que também examinaram a eficácia das mensagens de texto em vários ambientes de saúde^(20,26-27)^.

Pesquisadores dos EUA realizaram um estudo de intervenção piloto para avaliar o impacto das mensagens de texto na adesão à medicação entre adolescentes que receberam transplante de fígado. Os resultados indicaram que os participantes que receberam mensagens de texto, seja de elogio ou de cuidados habituais, apresentaram um aumento significativo na adesão às doses prescritas (OR: 2,49, *p* = 0,03), tomaram seus medicamentos conforme as instruções (OR: 2,39, *p* = 0,04) e demonstraram maior confiança no tratamento (OR: 2,46, *p*=0,04)^(20)^.

Um estudo brasileiro que avaliou a eficácia de mensagens de texto e aconselhamento telefônico na suspensão do tabagismo entre fumantes hospitalizados encontrou uma taxa de abstinência maior no grupo que recebeu mensagens telefônicas^(26)^.

Um ensaio clínico randomizado com 2.000 funcionários do sistema de saúde dos EUA, que analisou o efeito das mensagens de texto na adesão à vacinação contra a COVID-19, mostrou um aumento significativo de 4,9 pontos percentuais (IC 95% 0,8-9,1) na adesão no grupo de intervenção em comparação com o grupo controle (*p* = 0,02) após duas semanas^(27)^.

O uso de mensagens de texto em populações vulneráveis demonstrou apresentar benefícios socioculturais e econômicos significativos. Do ponto de vista sociocultural, as mensagens de texto aumentam o acesso à informação em saúde e melhoram a adesão às medidas preventivas, respeitando particularidades locais e culturais. Do ponto de vista econômico, elas oferecem uma solução econômica para a disseminação de informação e podem ajudar a reduzir custos de saúde ao promover uma melhor adesão ao tratamento e evitar complicações de saúde. Estes resultados destacam o potencial das mensagens de texto em desempenhar um papel fundamental nas estratégias de saúde pública, especialmente em contextos de vulnerabilidade^(28)^.

No presente estudo, houve uma melhora significativa na adesão às medidas de proteção individual na sexta e sétima semanas em comparação com a pontuação obtida antes da intervenção. A literatura sugere que a melhora da adesão geralmente ocorre entre o primeiro e o terceiro mês de intervenção^(27,29-31)^, o que é consistente com os resultados encontrados.

Não há consenso na literatura sobre a duração ideal da intervenção e a frequência de envio das mensagens, que pode variar de diária a cada três dias, por períodos de duas semanas a seis meses^(12,32)^. A escolha do tempo de intervenção e do número de mensagens dependerá do resultado a ser medido^(33-34)^. Entretanto, os estudos destacam que, independentemente da duração e da frequência, a intervenção deve ser atraente e não tediosa, para evitar o abandono das recomendações^(34-35)^.

Ao analisar cada questão individualmente, observou-se que a medida de proteção individual que teve adesão mais precoce foi o distanciamento social (distância de um metro das demais pessoas) ao sair de casa, que teve uma melhora significativa ao final da primeira semana. Acredita-se que este comportamento possa estar ligado ao fato de a população perceber esta medida como protetora, além de ser uma ação mais direta e fácil de ser implementada. Um estudo transversal realizado com 2.013 adultos nos EUA e na Europa mostrou que as motivações para o distanciamento social incluíam proteger a todos (86%), proteger a si mesmo (84%) e um senso de responsabilidade para proteger a comunidade (84%)^(36)^.

Por outro lado, quatro recomendações de proteção individual não apresentaram mudanças significativas ao longo do tempo: usar máscara ao sair de casa, lavar a máscara com água e sabão ao chegar em casa, usar máscara no metrô, ônibus, trem ou carro quando outra pessoa estiver presente e higienizar as mãos ao tocar em objetos fora de casa. A falta de melhoria significativa no uso da máscara ao sair de casa pode estar associada à alta pontuação de adesão registrada desde a primeira avaliação, na qual esta medida apresentou a pontuação mais alta. Desde o início da pandemia, o uso de máscaras foi amplamente promovido e exigido pelos órgãos públicos como uma das principais medidas de controle da doença, resultando em maior conscientização e adesão por parte da população^(37)^.

No entanto, a recomendação com a menor pontuação desde a primeira avaliação foi o uso de máscaras no metrô, ônibus, trem ou carro quando outra pessoa estivesse presente. Este resultado pode ter sido influenciado pela falta da alternativa “não aplicável” para os participantes que se deslocavam a pé ou sozinhos no carro, o que pode ter levado muitos a marcar a opção “nunca”, reduzindo a pontuação desta pergunta. Portanto, este resultado deve ser interpretado com cautela.

Um estudo destaca que a pandemia de COVID-19 causou mudanças globais no transporte e nos motivos e frequências de viagens, em que foi observada uma queda significativa no volume de passageiros no transporte público^(38)^. Um estudo identificou que 90% dos entrevistados reduziram totalmente (47%) ou limitaram (44%) o uso do transporte público, principalmente devido à mudança de trabalho e/ou aulas escolares de presenciais para remotas, além do medo de contrair a doença^(39)^.

O fato de não ter havido um aumento significativo na adesão à lavagem das máscaras com água e sabão ao chegar em casa pode ser atribuído à falta de divulgação desta medida pelos órgãos governamentais e pela mídia. Além disso, podemos inferir que estes resultados estão relacionados à sobrecarga de trabalho durante o *lockdown*. Um estudo que analisou os desafios enfrentados pelos profissionais em trabalho remoto durante o *lockdown* identificou dificuldades relacionadas à família, como aumento das responsabilidades com a escola, cuidados com os filhos e tarefas domésticas; problemas relacionados ao trabalho, como longas horas e espaço inadequado^(40)^ e problemas de saúde mental, como sintomas de ansiedade e depressão, exaustão e esgotamento^(40-41)^.

A baixa adesão à higienização das mãos ao tocar em objetos fora de casa pode ser explicada pela falta de acesso ou de material adequado para realizar esta medida de proteção. Um estudo realizado na cidade de Woldia, no nordeste da Etiópia, observou que a maioria dos participantes (63,1%, n = 255 de 404) não praticava a higiene das mãos em todos os locais devido a restrições de acesso a água e produtos de higiene^(42)^.

Considerando os resultados obtidos, as mensagens de texto enviadas por telefone celular demonstraram ser uma ferramenta eficaz para a educação em vários contextos de saúde, incluindo a prevenção de doenças emergentes. Esta abordagem permite atingir grupos amplos de pessoas, contribuindo para a transmissão de informação relevante e oportuna e, consequentemente, para a melhoria da adesão ao tratamento e às medidas de proteção.

Os resultados apresentados podem servir de guia para gestores de saúde no desenvolvimento de programas de prevenção e políticas públicas para emergências em saúde similares à pandemia de COVID-19. Além disso, estes resultados podem ser usados para capacitar profissionais de saúde, especialmente da área de enfermagem, a elaborar estratégias de educação em saúde direcionadas a grupos mais suscetíveis à não conformidade, aumentando a eficácia das intervenções e promovendo maior adesão às medidas de proteção.

Este estudo tem algumas limitações. Não foi possível verificar se todos os participantes leram as mensagens. Além disso, como se tratava de uma doença emergente, não havia nenhum instrumento validado para avaliar a adesão às recomendações de proteção individual. Considerando a urgência das intervenções para melhorar o conhecimento e a adesão da população, o instrumento utilizado foi desenvolvido pelos pesquisadores do centro principal com base nas recomendações disponíveis na época e apenas refinado pelos outros pesquisadores do estudo. Ademais, o instrumento não foi testado com a população-alvo antes do início do estudo, o que pode ter afetado alguns dos resultados encontrados e discutidos anteriormente. A pergunta sobre o uso de máscaras no transporte público não contava com a alternativa “não aplicável”, o que pode ter afetado os resultados.

Variáveis contraditórias, como idade, nível educacional, renda e mudanças nas políticas públicas, não foram controladas neste estudo. A falta de controle sobre estas variáveis limita a capacidade de estabelecer relações causais diretas entre a intervenção e as mudanças observadas. Por fim, a perda de amostra observada pode ter afetado os resultados, limitando a generalização dos resultados. Portanto, os efeitos identificados devem ser interpretados com cautela. Apesar das limitações mencionadas, os resultados são relevantes e fornecem percepções importantes para políticas públicas e estratégias de comunicação em saúde.

## Conclusão

As mensagens enviadas por telefone celular foram eficazes na promoção da adesão às práticas de prevenção de COVID-19, especialmente após a sexta semana de envio. Esta ferramenta de ensino pode ser considerada para outras pandemias, devido ao seu baixo custo e à sua eficácia comprovada na melhoria do conhecimento e da adesão em vários contextos.

Os resultados apresentados podem servir de guia para os gestores de saúde no desenvolvimento de programas de prevenção e políticas públicas para emergências em saúde semelhantes à pandemia de COVID-19. Além disso, o pesquisador pode usar este protocolo para orientar a intervenção em estudos futuros. Entretanto, devido ao número significativo de perdas durante o período de acompanhamento, recomenda-se reduzir a duração do seguimento para quatro semanas.

Além disso, recomenda-se que estudos futuros avaliem as evidências de validade do instrumento desenvolvido e utilizem um projeto randomizado para fortalecer ainda mais os resultados.

## Data Availability

Todos os dados gerados ou analisados durante este estudo estão incluídos neste artigo publicado.

# Translation: Eficacia de los mensajes de texto en la adherencia a las prácticas de prevención el COVID-19: un estudio cuasi experimental*

**DOI:** 10.1590/1518-8345.7810.4706

**Authors:** Vinicius Lino de Souza, Sheila Coelho Ramalho Vasconcelos Morais, Rafaela Batista dos Santos Pedrosa, Mirian Ueda Yamaguchi, Eduesley Santana Santos, Agueda Maria Ruiz Zimmer Cavalcante, Vinícius Batista Santos, Juliana de Lima Lopes

**Affiliations:** 1 Universidade Federal de São Paulo, Escola Paulista de Enfermagem, São Paulo, SP, Brasil. Universidade Federal de São Paulo Escola Paulista de Enfermagem SP São Paulo Brasil; 2 Becario del Conselho Nacional de Desenvolvimento Científico e Tecnológico (CNPq), Brasil. Becario del Conselho Nacional de Desenvolvimento Científico e Tecnológico Brasil; 3 Universidade Federal do Pernambuco, Departamento de Enfermagem, Recife, PE, Brasil. Universidade Federal do Pernambuco Departamento de Enfermagem PE Recife Brasil; 4 Universidade Estadual de Campinas, Departamento de Enfermagem, Campinas, SP, Brasil. Universidade Estadual de Campinas Departamento de Enfermagem SP Campinas Brasil; 5 Centro Universitário de Maringá, Departamento de Saúde, Maringá, PR, Brasil. Centro Universitário de Maringá Departamento de Saúde PR Maringá Brasil; 6 Universidade Federal de Sergipe, Departamento de Enfermagem, Maceió, SE, Brasil. Universidade Federal de Sergipe Departamento de Enfermagem SE Maceió Brasil; 7 Universidade Federal do Goiás, Faculdade de Enfermagem, Goiânia, GO, Brasil. Universidade Federal do Goiás Faculdade de Enfermagem GO Goiânia Brasil

**Keywords:** Envío de Mensajes de Texto, Coronavírus, Aislamiento Social, Máscaras, Cumplimiento y Adherencia al Tratamiento, Terapia Conductista

## Abstract

analizar la efectividad de los mensajes enviados por teléfono móvil sobre la adherencia al uso de mascarillas, la higiene de manos y el distanciamiento social durante la pandemia de COVID-19.

ensayo clínico cuasi experimental multicéntrico realizado en cuatro universidades brasileñas entre julio y octubre de 2020. La muestra consistió en 429 personas de diferentes ciudades brasileñas que recibieron mensajes cada dos días durante siete semanas sobre medidas de protección personal. La adherencia a las medidas se analizó mediante un instrumento desarrollado por los investigadores del centro principal y perfeccionado por los demás investigadores, compuesto por 13 preguntas y una escala de Likert de cinco puntos, con la puntuación global entre 13 y 65 puntos, donde a mayor puntuación, mayor adherencia. El análisis estadístico se realizó mediante el análisis multivariante de la varianza (MANOVA), considerando*p*< 0,05 como significativo.

se observó una mejora en la puntuación de la adherencia a lo largo de las semanas. En comparación con la puntuación obtenida antes de la intervención, se observó una mejora significativa de la adherencia en las dos últimas semanas (*p*< 0,001).

la intervención mediante mensajes enviados por teléfono móvil demostró ser eficaz para promover la adherencia a las prácticas de protección individual contra el COVID-19. Sin embargo, la pérdida de muestra observada puede haber afectado a los resultados, limitando su generalización. Por lo tanto, los efectos identificados deben interpretarse con cautela. *Registro Brasileiro de Ensaios Clínicos* (RBR-2s94rb).

## Destacados

**(1)** Uso de tecnología de bajo coste para promover la educación en salud. **(2)** Estrategia para reforzar o mejorar las políticas en salud. **(3)** El rol de los profesionales de enfermería como organizadores de la estrategia de adherencia.

## Introducción

El COVID-19, causado por el virus SARS-CoV-2, fue identificado como un problema de salud mundial y declarado pandemia por la Organización Mundial de la Salud (OMS) el 11 de marzo de 2020^(1)^. Aunque el COVID-19 ya no está clasificado como pandemia, todavía se observan casos de hospitalización y muertes relacionadas con la enfermedad^(2-3)^.

Al principio de la pandemia, los gobiernos de varios países adoptaron una serie de estrategias para contener la propagación del SARS-CoV-2 y mitigar sus repercusiones en la salud y la economía, como la realización de pruebas en la comunidad, la detección de contactos, el aislamiento y las medidas de protección personal^(4)^.

Las recomendaciones para la protección personal estaban relacionadas con el uso y la retirada correctos de mascarillas, una distancia social de un metro entre las personas y la higiene de las manos con agua y jabón o alcohol al 70%^(5-6)^. Estas medidas no eran prácticas habituales entre la población brasileña, lo que suponía un reto aún mayor para los gobiernos del país. Los investigadores señalaron que, debido a la alta transmisibilidad del virus y a la falta de tratamiento farmacológico y de vacunación contra el COVID-19 al inicio de la pandemia, el control de la enfermedad sólo sería posible mediante estos cambios de comportamiento masivos y rápidos^(7)^.

Otro factor que se convirtió en un reto al inicio de la pandemia fue la escasez de mascarillas quirúrgicas, por lo que la OMS recomendó que estas mascarillas se reservaran para el personal sanitario, las personas con síntomas de la enfermedad y los cuidadores o quienes compartieran el mismo espacio con personas con sospecha de COVID-19 o síntomas respiratorios^(5)^.

La OMS también ha afirmado que los responsables de la toma de decisiones de cada país deberían asesorar sobre el uso de mascarillas no quirúrgicas en relación con el número de capas y los tipos de tejido, así como la forma y el ajuste de la mascarilla^(5)^. Teniendo en cuenta estas recomendaciones, la *Agência Nacional de Vigilancia Sanitária* de Brasil (ANVISA) recomendó que las mascarillas no quirúrgicas constaran de tres capas de tejido, siendo la capa exterior de tejido no impermeable, la capa central de tejido transpirable y la capa interior de tejido de algodón^(6)^.

A pesar de la necesidad de tantos cambios en la rutina diaria de la población, algunos estudios han identificado que la población deseaba practicar medidas de protección individual contra el COVID-19 y que el cumplimiento de estas recomendaciones era mayor cuando estaba asociado a un mayor conocimiento^(8-9)^.

Un estudio transversal realizado en Arabia Saudí con 502 participantes adultos mayores de 65 años o más mostró que más de la mitad de los participantes (52,2%) tenían intención de practicar medidas de protección personal contra el COVID-19. Además, las probabilidades de poner en práctica las recomendaciones preventivas eran 1,59 veces (IC 95% = 1,01-2,52) mayores en las mujeres que en los hombres y 2,72 veces (IC 95% = 1,44-5,16) mayores en las personas con un alto nivel de estudios^(8)^.

Se obtuvieron resultados similares en Estados Unidos de América (EE.UU), donde una encuesta realizada a 506 residentes reveló que los participantes que percibían como eficaces las prácticas sanitarias recomendadas por los Centros para el Control y la Prevención de Enfermedades de Estados Unidos de América (CDC) tenían mayores intenciones de llevarlas a cabo. Además, la percepción de los participantes sobre la gravedad del COVID-19 en Estados Unidos de América fue un factor significativo (β = 0,48; *p* ≤ 0,001; 95% IC = 0,418–0,539). La eficacia percibida de las prácticas recomendadas fue responsable de la mayor variación en las intenciones de comportamiento (β = 0,19; *p*≤0,001; 95% IC=0,112–0,260)^(9)^.

En este sentido, las intervenciones de educación sanitaria para mejorar el conocimiento y la adherencia de la población a las medidas de protección individual se han convertido en esenciales y fundamentales para el control de la enfermedad. Las acciones de educación para la salud son una práctica cotidiana realizada por los profesionales de enfermería y se pueden adoptar diversas estrategias, como el envío de mensajes^(10-11)^.

Un metaanálisis en el que se evaluaron nueve ensayos clínicos aleatorizados con 1.121 participantes mostró un impacto favorable de la mensajería por teléfono móvil en comparación con la atención habitual sobre la adherencia a la medicación entre individuos con diabetes mellitus tipo 2 (diferencia de medias estandarizada: 0,36; 95% IC: 0,14-0,59)^(12)^.

En un ensayo clínico aleatorizado se evaluó si los estímulos conductuales enviados a través de mensajes de texto podían acelerar el cumplimiento de la política de vacunación contra el COVID-19 de un sistema sanitario entre 2.000 empleados de sistemas sanitarios del medio oeste o el sur de EE.UU. que no cumplían la política de vacunación un mes antes de la fecha límite. Los resultados revelaron que, al final de la intervención de dos semanas, 363 participantes del grupo incentivado por mensajes de texto (36,3%) y 318 participantes del grupo de control (31,8%) se habían adherido a la política de vacunación, lo que representaba un aumento significativo de 4,9 puntos porcentuales (IC 95%, 0,8 a 9,1; *p*=0,002) en los análisis ajustados que comparaban el grupo incentivado con el grupo de control. Entre los participantes que se adhirieron al final del periodo de seguimiento de cuatro semanas, el incentivo por mensaje de texto redujo significativamente el tiempo de adhesión en una media de 2,4 días (95% IC, 2,1 a 4,7; *p*<0,001) y una mediana de 5,0 días (95% IC, 2,5 a 7,7; *p*<0,001), en comparación con el grupo de control^(13)^.

Estos resultados están en consonancia con otro metanálisis que analizó el efecto de los mensajes para controlar el exceso de peso e incluyó 12 ensayos controlados aleatorios. Diez de los estudios incluidos mostraron un efecto significativo en la pérdida de peso de los participantes y la diferencia media agrupada en el cambio del índice de masa corporal tras la intervención fue de -0,43 kg/m² (95% IC -0,63 a -0,23 kg/m²^(14)^.

En Brasil, la mensajería de texto es un medio de comunicación muy utilizado, especialmente en regiones donde el acceso a Internet es limitado o donde el uso de teléfonos inteligentes avanzados aún no está generalizado^(15)^. Este recurso es especialmente valioso en contextos de salud pública, ya que facilita la rápida difusión de información y llega a diversos segmentos de la población, incluidos aquellos con niveles más bajos de educación o acceso limitado a otras fuentes de información^(15)^. Además, los factores culturales, como la confianza en la comunicación directa, pueden afectar a la forma en que se reciben los mensajes y al grado de adhesión a las directrices^(16)^.

Aunque existen investigaciones sobre campañas de salud pública y estrategias de persuasión, la evidencia sobre cómo estos mensajes influyen específicamente en las poblaciones durante crisis sanitarias a gran escala, como la pandemia de COVID-19, sigue siendo limitada. Además, aunque en la bibliografía se documentan diversos enfoques persuasivos, incluidos los mensajes pro sociales, la prueba social y los llamamientos morales, pocos estudios han explorado el impacto de estas estrategias en el contexto de las enfermedades infecciosas emergentes, especialmente teniendo en cuenta la politización que ha marcado la pandemia de COVID-19. Por lo tanto, es esencial investigar cómo esta estrategia ha afectado a la adherencia a las conductas preventivas en un contexto tan singular.

Por ello, el objetivo de este estudio fue analizar la eficacia de los mensajes enviados por teléfono móvil sobre la adherencia al uso de mascarillas, la higiene de manos y el distanciamiento social durante la pandemia de COVID-19.

## Método

### Diseño del estudio

Se trata de un estudio clínico cuasi experimental y multicéntrico realizado según las directrices del *Transparent Reporting of Evaluations with Non-randomized Designs*(TREND).

### Ubicación

Este estudio se llevó a cabo en cuatro universidades públicas de los estados brasileños de São Paulo, Paraná, Pernambuco y Sergipe.

### Período

La recogida de datos tuvo lugar entre julio y octubre de 2020.

### Población y criterios de selección

La población del estudio estuvo formada por adultos brasileños. Los criterios de elegibilidad incluyeron: vivir en Brasil, tener 18 años o más, tener un teléfono móvil y acceso a Internet para utilizar la aplicación *WhatsApp*®, no tener discapacidad visual y/o cognitiva, saber leer y escribir y dominar el idioma portugués.

### Definición de la muestra

El tamaño de la muestra se calculó con el programa informático *G Power*®, versión 3.1.9.2 (disponible en https://www.gpower.hhu.de/). Sobre la base de un tamaño del efecto de Cohen de 0,15, una potencia de prueba de 0,80 y un nivel de significación del 5%, se determinó que la muestra mínima necesaria era de 351 participantes. Para compensar las posibles pérdidas durante el seguimiento, se añadió un 20% al tamaño de la muestra, lo que dio como resultado un tamaño mínimo de 421 participantes.

### Intervenciones

La intervención consistió en el envío de mensajes previamente validados^(17)^. El contenido de los mensajes de texto se elaboró mediante una revisión narrativa de la literatura, con el objetivo de analizar las recomendaciones relacionadas con el uso de mascarilla, la higiene de las manos y el distanciamiento social durante la pandemia de SARS-CoV-2.

Se crearon 18 mensajes que abarcaban temas como la enfermedad, los métodos de transmisión y las medidas preventivas relacionadas con el distanciamiento social, el uso de mascarillas y la higiene de las manos. Estos mensajes se diseñaron utilizando frases concisas y un tono orientado a la acción para promover la adopción de medidas preventivas contra el COVID-19, acompañados de imágenes ilustrativas relevantes para cada tema^(17)^. A continuación, se muestra un ejemplo del texto de uno de los mensajes elaborados: “*La mascarilla es una medida de protección frente al coronavirus, y debes llevarla siempre puesta cuando salgas de casa*”^(17)^.

Tras su elaboración, los mensajes fueron evaluados por un grupo de expertos, que los examinaron en función de su pertinencia teórica, claridad, aplicabilidad práctica y vocabulario. La validez del contenido se comprobó calculando el Índice de Validez del Contenido (IVC), siendo necesarias dos rondas de evaluación para que los mensajes alcanzaran un IVC superior al 90%, lo que garantizaba su validez^(17)^.

Estos mensajes se enviaron por teléfono móvil dos o tres veces por semana, con un intervalo de dos días entre cada envío, durante siete semanas. La decisión de utilizar un intervalo de dos días se basó en un estudio previo que examinó el impacto del envío de mensajes de texto con esta frecuencia, centrándose en la adherencia y el autocuidado en individuos con enfermedad arterial coronaria. En este estudio, la mayoría de los participantes se mostraron satisfechos con la recepción de mensajes con un intervalo de dos días^(18)^. No existe consenso en la literatura sobre la frecuencia ideal para el envío de mensajes, con intervalos que van de diarios a semanales o quincenales^(12,19)^. El período de siete semanas para la intervención se adoptó sobre la base de estudios que utilizaron esta estrategia de educación sanitaria con otras poblaciones y que emplearon períodos de seguimiento similares^(20-21)^.

### Variables e instrumentos

La adherencia se evaluó mediante un instrumento desarrollado inicialmente por investigadores del centro principal del estudio y perfeccionado posteriormente por ocho profesionales sanitarios con un máster o doctorado en enfermedades transmisibles o infecciosas. Cada experto tenía al menos tres años de experiencia en enfermedades infecciosas o en la gestión de situaciones relacionadas con pandemias. El proceso de perfeccionamiento se llevó a cabo a través de una reunión virtual moderada por el supervisor del estudio. Durante la sesión, se revisó cuidadosamente cada punto del instrumento, con continuas discusiones hasta que los expertos llegaron a un consenso.

Este instrumento, que se ajusta a los mensajes utilizados en la intervención, contiene 13 preguntas sobre el uso correcto de las mascarillas, la importancia de la higiene de las manos y el distanciamiento social. Cada ítem del instrumento tiene una escala Likert de cinco puntos (1= Nunca, 2= Rara vez, 3= A veces, 4= Casi siempre, 5= Siempre), en la que la puntuación global oscila entre 13 y 65 puntos, y a mayor puntuación, mayor adherencia. Tres preguntas de este instrumento tienen puntuaciones invertidas: 1) En la última semana, ¿con qué frecuencia se quitó la mascarilla para hablar por teléfono cuando estaba fuera de casa? 2) En la última semana, ¿con qué frecuencia se quitó la mascarilla para ir al baño cuando estaba fuera de casa? 3) En la última semana, ¿con qué frecuencia se quitó la mascarilla para hablar con alguien cuando estaba fuera de casa? Estas preguntas se puntuaron de la siguiente manera: Nunca = 5 puntos, Rara vez = 4 puntos, A veces = 3 puntos, Casi siempre = 2 puntos y Siempre = 1 punto. El instrumento se aplicó antes del inicio de las intervenciones y después semanalmente, una vez que los mensajes habían empezado a transmitirse.

### Recogida de datos

El protocolo del estudio siguió los siguientes pasos: El reclutamiento de los individuos se realizó mediante una invitación enviada vía *WhatsApp*® desde los contactos de los investigadores en todos los centros, con la petición de publicitarlo en otros grupos. La invitación incluía información sobre los criterios de inclusión adoptados.

A los participantes que aceptaron participar en el estudio se les pidió en primer lugar que confirmaran que cumplían los criterios de inclusión y, a continuación, firmaron electrónicamente el Formulario de Consentimiento Informado. A continuación, rellenaron el formulario de inscripción (inscripción inicial), que se puso a su disposición a través de la plataforma *Google Forms*®. El mensaje inicial se envió al día siguiente, y los mensajes posteriores se enviaron a intervalos de dos días (de dos a tres veces por semana) durante un periodo de siete semanas, con un total de 18 mensajes por participante.

Los datos se analizaron semanalmente, todos los domingos. Los participantes que no completaron las siete semanas de intervención o que se retiraron del estudio fueron excluidos del análisis final.

### Análisis de los datos

Los datos se introdujeron inicialmente en *Microsoft Excel*® versión 2020 y se utilizó el programa estadístico SPSS® versión 25.0 para analizarlos. Inicialmente, se realizaron estadísticas descriptivas, evaluando medidas de tendencia central y frecuencia.

La prueba de Kolmogorov-Smirnov proporcionó evidencias de que los ítems del instrumento no siguen una distribución normal. Sin embargo, como los datos procedían de una muestra suficientemente amplia, basándose en el teorema del límite central, se supuso que el supuesto de normalidad no afectaba al análisis de los resultados^(22)^. Por lo tanto, se utilizaron pruebas estadísticas paramétricas para evaluar la adherencia a las medidas de protección a lo largo de las semanas. Además, cuando se confirmó la homogeneidad de los datos, se realizaron pruebas ANOVA o MANOVA, teniendo en cuenta la esfericidad de los datos. Un valor *p*< 0,05 se consideró significativo.

### Aspectos éticos

El estudio fue aprobado por el Comité de Ética en Investigación, dictamen número 4.077.371, y fue registrado en el *Registro Brasileiro de Ensaios Clínicos* (ReBEC), númeroRBR-2s94rb.

## Resultados

Se incluyeron en el estudio 820 participantes. Fue necesario aumentar el tamaño de la muestra calculada debido a la identificación, durante el seguimiento, de un número muy elevado de abandonos del estudio, probablemente asociados a la sobrecarga provocada por la pandemia. Durante el seguimiento se perdieron 391 participantes, con un total de 429 individuos analizados (Figura 1).

**Figura 1-:** Diagrama de flujo adaptado de Transparent Reporting of Evaluations with Nonrandomized Designs (TREND)^(23)^

Para mitigar el impacto de las pérdidas de muestras, se realizó un análisis *post hoc* de la potencia estadística basado en la muestra final de 429 participantes. Se observó un tamaño del efecto d de Cohen de 0,185, una potencia de prueba de 0,80 y un nivel de significación del 5%, lo que demuestra que el estudio tiene potencia suficiente para detectar los efectos esperados.

Los participantes reclutados de las cuatro universidades brasileñas procedían de 113 ciudades diferentes de 21 estados brasileños (Acre, Alagoas, Amazonas, Bahia, Ceará, Distrito Federal, Goiás, Maranhão, Mato Grosso do Sul, Minas Gerais, Pará, Paraná, Paraíba, Pernambuco, Roraima, Rio de Janeiro, Río Grande do Norte, Río Grande do Sul, Santa Catarina, São Paulo y Sergipe).

Los participantes tenían una edad media de 31,02±10,88 años y un número medio de hijos de 1,82±0,85. La Tabla 1 muestra las demás características sociodemográficas de los participantes en el estudio.

**Tabla 1-:** Características sociodemográficas de los participantes (n = 429). São Paulo, SP, Brasil, 2020

**Variable**	n* (%) ^†^
Sexo: Femenino	327 (76,22)
Edad: hasta 30 años	245 (57,11)
Estado civil	
Soltero (a)	221 (51,51)
Casado (a)/Unión estable	176 (41,03)
Divorciado (a)	28 (6,53)
Viudo (a)	4 (0,93)
Ingresos familiares	
Menos de un salario-mínimo ^‡^	25 (5,83)
De uno a tres salarios-mínimos ^‡^	114 (26,57)
De cuatro a siete salarios-mínimos ^‡^	138 (32,17)
Más de siete salarios-mínimos ^‡^	152 (35,43)
Tiene hijos	163 (38,00)
Años de escolaridad	
Menos de nueve años de escolaridad	62 (14,46)
Entre nueve y once años de escolaridad	172 (40,09)
Más de once años de escolaridad	195 (45,45)
Religión	
Católico (a)	157 (36,60)
Evangélico (a)	99 (23,07)
No religioso (a)	94 (21,91)
Espiritista	47 (10,96)
Adepto (a) de *Umbanda*	16 (3,73)
Cristiano (a)	7 (1,63)
Agnóstico (a)	5 (1,16)
Testigo de Jehová	2 (0,47)
Hermético (a)/Sabático (a)	2 (0,47)
Número de convivientes	
0	51 (11,89)
1	88 (20,51)
2	104 (24,24)
3	96 (22,38)
4	62 (14,45)
Más de 4 personas	28 (6,53)
Empleado (a)	260 (60,61)
Empleado (a) registrado (a)	231 (88,84)
Empleado (a) no registrado (a)	29 (11,16)

^*^n = Número de personas; ^†^% = Porcentaje;^‡^Salario-mínimo = R$ 1.039,00 (BRL) en 2020

Al analizar la adherencia a las medidas de protección individuales frente al COVID-19, la Figura 2 y la Tabla 2 muestran que se produjo un aumento progresivo de la puntuación de adherencia a lo largo de las evaluaciones. Además, se produjo una mejora significativa en las dos últimas semanas en comparación con la puntuación obtenida antes de la intervención.

**Figura 2-:** Puntuación mediana de la adherencia de los participantes durante las evaluaciones (n = 429). São Paulo, SP, Brasil, 2020

**Tabla 2-:** Análisis de las puntuaciones de adherencia de los participantes durante las evaluaciones (n = 429). São Paulo, SP, Brasil, 2020

**Semana de evaluación**	**Mínimo**	**Máximo**	**25%**	**Mediana**	**75%**	QI*	**Media**	**95% IC** ^†^	**DE** ^‡^	**CV** ^§^	**Valor *p*** ^||^	**Tamaño del efecto** ^¶^	**Valor *p*** ^**^
Antes de la intervención	22,00	65,00	51,00	57,00	60,00	9,00	54,59	53,85; 55,33	7,81	14,31	<0,001	0,022	<0,001
Primera semana	25,00	65,00	53,00	57,00	61,00	8,00	55,63	54,94; 56,32	7,24	13,01	<0,001		
Segunda semana	25,00	65,00	52,00	57,00	61,00	9,00	55,88	55,21; 56,56	7,10	12,71	<0,001		
Tercera semana	25,00	65,00	53,00	58,00	61,00	8,00	55,96	55,26; 56,66	7,35	13,14	<0,001		
Cuarta semana	23,00	65,00	53,00	58,00	61,00	8,00	56,22	55,48; 56,95	7,74	13,77	<0,001		
Quinta semana	25,00	65,00	54,00	58,00	61,00	7,00	56,77	56,12; 57,42	6,89	12,13	<0,001		
Sexta semana	20,00	65,00	54,00	59,00	61,00	7,00	57,03 ^††^	56,38; 57,68	6,85	12,02	<0,001		
Séptima semana	28,00	65,00	55,00	59,00	62,00	7,00	57,39 ^††^	56,79; 57,99	6,32	11,02	<0,001		

*QI = Rango interquartil; ^†^IC = Intervalo de Confianza; ^‡^DE = Desviación estándar; ^§^CV = Coeficiente de Variación; ^||^Prueba de Kolmogorov-Smirnov para medir la normalidad de los datos; ^¶^Eta cuadrado parcial; **Prueba MANOVA; ^††^Medias con diferencias estadísticamente significativas respecto a los valores basales tras aplicar la prueba de Bonferroni

Utilizando el alfa de Cronbach para evaluar la consistencia interna de los datos, el instrumento mostró un alfa global superior a 0,70, con la excepción de la pregunta 1, que tuvo un alfa de 0,62. Las preguntas 06, 09, 10, 11 y 12 tuvieron valores de alfa que oscilaron entre 0,74 y 0,78, mientras que las preguntas 02, 03, 04, 05, 07, 08 y 13 presentaron la mayor consistencia interna con valores alfa entre 0,80 y 0,90.

Al analizar cada pregunta individualmente y compararlas con la puntuación de adherencia obtenida antes del inicio de la intervención, la Tabla 3 muestra que la medida de protección individual con la primera mejora fue la distancia de un metro de las personas al salir de casa, mostrando una mejora significativa al final de la primera semana. Las recomendaciones de higienizarse las manos al tocar la parte frontal de la mascarilla y de quitarse la mascarilla por la banda elástica tras su uso mostraron una mejora significativa en la tercera y cuarta semanas, respectivamente.

En la quinta semana, se observaron progresos significativos en las medidas relacionadas con cambiarse la mascarilla cuando estaba mojada, mantener la mascarilla puesta cuando se hablaba con alguien fuera de casa y hablar por teléfono fuera de casa. En la sexta semana, se observaron mejoras significativas en la medida de llevar la mascarilla puesta al ir al baño fuera de casa y llevar una mascarilla adicional en el bolso para cambiársela. La medida de llevar la mascarilla cubriendo la boca y la nariz mostró una mejora significativa sólo en la séptima semana.

Por último, cuatro medidas no mostraron cambios significativos a lo largo del tiempo: llevar la mascarilla al salir de casa; lavarse la mascarilla con agua y jabón al llegar a casa; llevar la mascarilla en el metro, el autobús, el tren o el coche con otra persona presente; y desinfectarse las manos al tocar objetos fuera de casa.

**Tabla 3-:** Análisis de las puntuaciones de adhesión de los participantes a cada una de las preguntas del instrumento a lo largo de las evaluaciones (n = 429). São Paulo, SP, Brasil, 2020

Preguntas	Antes de la intervención	Primera semana	Segunda semana	Tercera semana	Cuarta semana	Quinta semana	Sexta semana	Séptima semana	Valor
	Media (DE*)	Media (DE*)	Media (DE*)	Media (DE*)	Media (DE*)	Media (DE*)	Media (DE*)	Media (DE*)	*p* ^†^
1. En la última semana, ¿con qué frecuencia utilizó la mascarilla al salir de casa?	4,87 (0,47)	4,86 (0,47)	4,85 (0,47) ^‡^	4,85 (0,50)	4,8 (0,64)	4,85 (0,56)	4,89 (0,46)	4,93 (0,28) ^‡^	0,004
2. En la última semana, ¿con qué frecuencia lavó la mascarilla con agua y jabón al llegar a casa?	4,10 (1,34)	4,21 (1,24)	4,18 (1,26)	4,16 (1,26)	4,15 (1,30)	4,14 (1,29)	4,26 (1,20)	4,26 (1,21)	0,183
3. En la última semana, ¿con qué frecuencia se quitó la mascarilla para hablar por teléfono cuando estaba fuera de casa?	4,35 (1,14)	4,42 (1,10)	4,48 (0,99)	4,48 (1,01)	4,48 (1,06)	4,57 (0,93) ^§^	4,56 (0,95) ^§^	4,57 (0,95) ^§^	<0,001
4. En la última semana, ¿con qué frecuencia te quitaste la mascarilla para ir al baño cuando estaba fuera de casa?	4,38 (1,11)	4,42 (1,09)	4,48 (1,02)	4,46 (1,06)	4,49 (0,99)	4,53 (0,91)	4,56 (0,88) ^§^	4,56 (0,88) ^§^	0,004
5. En la última semana, ¿con qué frecuencia ha llevado mascarilla en el metro, el autobús, el tren o el coche en presencia de otras personas?	3,36 (1,78)	3,48 (1,73)	3,49 (1,70)	3,39 (1,75)	3,45 (1,75)	3,45 (1,75)	3,53 (1,75)	3,49 (1,76)	0,327
6. En la última semana, ¿con qué frecuencia te has cambiado la mascarilla cuando estaba mojada?	3,95 (1,43)	4,09 (1,35)	4,12 (1,31)	4,11 (1,27)	4,19 (1,25)	4,21 (1,20) ^§^	4,21 (1,22) ^§^	4,32 (1,08) ^§^	<0,001
7. En la última semana, ¿con qué frecuencia te has quitado la mascarilla para hablar con alguien cuando estabas fuera de casa?	4,38 (1,05)	4,43 (1,02)	4,43 (1,03)	4,43 (1,07)	4,52 (1,00)	4,55 (0,93) ^§^	4,54 (0,95) ^§^	4,59 (0,86) ^§^	<0,001
8. En la última semana, ¿con qué frecuencia te has puesto una mascarilla que te cubriera la boca y la nariz cuando estabas fuera de casa?	4,71 (0,82)	4,76 (0,72)	4,76 (0,70)	4,74 (0,75)	4,76 (0,75)	4,81 (0,63)	4,83 (0,64)	4,86 (0,55) ^§^	<0,001
9. En la última semana, ¿con qué frecuencia ha llevado una mascarilla adicional en el bolso para cambiarla en caso necesario?	3,90 (1,48)	4,05 (1,38)	4,07 (1,37)	4,12 (1,33)	4,13 (1,31)	4,12 (1,37)	4,15 (1,31)	4,15 (1,30)	0,024
10. En la última semana, ¿con qué frecuencia mantuviste una distancia de un metro con otras personas cuando salías de casa?	3,89 (1,13)	4,09 (0,96) ^§^	4,13 (0,97) ^§^	4,11 (1,03) ^§^	4,16 (1,04) ^§^	4,18 (1,0) ^§^	4,22 (0,92) ^§^	4,24 (0,93) ^§^	<0,001
11. En la última semana, ¿con qué frecuencia se ha quitado la mascarilla por los bucles de las orejas después de usarla?	4,35 (1,08)	4,48 (0,95)	4,50 (0,95)	4,53 (0,97)	4,54 (0,94) ^§^	4,65 (0,79) ^§^	4,65 (0,79) ^§^	4,68 (0,72) ^§^	<0,001
12. En la última semana, ¿con qué frecuencia se higienizó las manos o utilizó alcohol al 70% después de tocar la parte delantera de la mascarilla?	4,05 (1,14)	4,02 (1,13)	4,08 (1,14)	4,22 (1,03) ^§^	4,21 (1,07) ^§^	4,32 (0,95) ^§^	4,28 (0,98) ^§^	4,30 (0,97) ^§^	<0,001
13. En la última semana, ¿con qué frecuencia se ha desinfectado las manos o ha utilizado alcohol al 70% después de tocar un objeto fuera de casa?	4,31 (0,96)	4,32 (0,93)	4,31 (0,96)	4,36 (0,96)	4,35 (0,95)	4,40 (0,88)	4,38 (0,95)	4,43 (0,88)	0,150

*DE = Desviación estándar; ^†^Prueba MANOVA; ^‡^Medias con diferencias estadísticamente significativas entre semanas tras aplicar la prueba de Bonferroni; ^§^Medias con diferencias estadísticamente significativas en comparación con los valores basales tras aplicar la prueba de Bonferroni

## Discusión

Este estudio cuasi experimental tuvo como objetivo evaluar la eficacia de los mensajes de texto enviados a través de teléfonos móviles en la promoción de medidas de protección individual contra el COVID-19 entre los brasileños de diferentes regiones. A pesar de las limitaciones inherentes a este diseño de estudio, como la ausencia de un grupo de control aleatorizado, que podría afectar a la validez interna de los resultados, los investigadores optaron por este enfoque debido a consideraciones éticas. Durante el periodo de recogida de datos, Brasil se enfrentaba a importantes discrepancias gubernamentales en la respuesta a la pandemia de COVID-19, lo que dejaba a la población sin directrices claras sobre cómo proceder^(24)^.

El análisis retórico de la comunicación de riesgos durante la pandemia de COVID-19 en Brasil revela una importante desorganización y mensajes contradictorios por parte de las autoridades nacionales y de los estados. Este enfoque creó una incertidumbre generalizada y socavó la adhesión a las medidas preventivas, como el distanciamiento social, promovidas de forma incoherente por la Presidencia de la República y el Ministerio de Salud. Además, los frecuentes cambios de ministros de salud y la creciente polarización política han socavado la coordinación de las políticas de salud pública, afectando gravemente a la respuesta del país a la pandemia^(25)^.

A nivel de los estados, sin embargo, la comunicación de riesgos se ajustaba más a las directrices de la OMS, abogando por medidas como el distanciamiento social horizontal. Sin embargo, los estados también se enfrentaron a importantes retos, como un sistema de pruebas ineficaz y la escasez de recursos. Los mensajes contradictorios entre los gobiernos federal y estaduales crearon un ambiente de inseguridad, dejando a la población, especialmente a la más vulnerable, insegura sobre las mejores formas de protegerse^(25)^.

Esta incertidumbre ha creado un terreno fértil para la propagación de noticias falsas y crisis políticas. En consecuencia, cuanto mayor sea el acceso a la información basada en las mejores pruebas científicas, mayor será la adherencia a las medidas de prevención del COVID-19 y, en última instancia, menor será la incidencia y la mortalidad asociadas a la enfermedad.

Los resultados indicaron una mejora de la adherencia a lo largo de las evaluaciones. Se han comunicado resultados similares en otros estudios que también han examinado la eficacia de los mensajes de texto en diversos entornos sanitarios^(20,26-27)^.

Investigadores de los Estados Unidos de América llevaron a cabo un estudio piloto de intervención para evaluar el impacto de los mensajes de texto en la adherencia a la medicación entre adolescentes que habían recibido un trasplante de hígado. Los resultados indicaron que los participantes que recibieron mensajes de texto ya fueran complementarios o de atención habitual, mostraron un aumento significativo del cumplimiento de las dosis prescritas (OR: 2,49, *p* = 0,03), tomaron la medicación según las instrucciones (OR: 2,39, *p* = 0,04) y mostraron mayor confianza en el tratamiento (OR: 2,46, *p*=0,04)^(20)^.

Un estudio brasileño que evaluó la eficacia de los mensajes de texto y el asesoramiento telefónico para dejar de fumar entre fumadores hospitalizados constató una mayor tasa de abstinencia en el grupo que recibió mensajes telefónicos^(26)^.

Un ensayo clínico aleatorizado con 2.000 trabajadores sanitarios estadounidenses, que analizó el efecto de los mensajes de texto en la adherencia a la vacunación COVID-19, mostró un aumento significativo de 4,9 puntos porcentuales (IC 95% 0,8-9,1) adherencia en el grupo de intervención en comparación con el grupo de control (*p* = 0,02) después de dos semanas^(27)^.

El uso de mensajes de texto en poblaciones vulnerables ha demostrado tener importantes beneficios socioculturales y económicos. Desde el punto de vista sociocultural, los mensajes de texto aumentan el acceso a la información sanitaria y mejoran la adherencia a las medidas preventivas, respetando al mismo tiempo las particularidades locales y culturales. Desde el punto de vista económico, ofrecen una solución rentable para difundir información y pueden ayudar a reducir los costes sanitarios al promover una mejor adherencia al tratamiento y evitar complicaciones de salud. Estos resultados ponen de relieve el potencial de los mensajes de texto para desempeñar un papel clave en las estrategias de salud pública, especialmente en contextos de vulnerabilidad^(28)^.

En el presente estudio, hubo una mejora significativa en la adherencia a las medidas de protección personal en la sexta y séptima semanas en comparación con la puntuación obtenida antes de la intervención. La bibliografía sugiere que las mejoras en la adherencia generalmente se producen entre el primer y el tercer mes de la intervención^(27,29-31)^, lo que está de acuerdo con los resultados encontrados en este estudio.

No existe consenso en la literatura sobre la duración ideal de la intervención y la frecuencia de envío de los mensajes, que puede variar de diaria a cada tres días, durante periodos de dos semanas a seis meses^(12,32)^. La elección del tiempo de intervención y del número de mensajes dependerá del resultado que se desee medir^(33-34)^. Sin embargo, los estudios destacan que, independientemente de la duración y la frecuencia, la intervención debe ser atractiva y no tediosa, para evitar el abandono de las recomendaciones^(34-35)^.

Al analizar cada cuestión individualmente, se observó que la medida de protección individual que tuvo una adherencia más temprana fue el distanciamiento social (alejarse un metro de otras personas) al salir de casa, que mejoró significativamente al final de la primera semana. Se cree que este comportamiento puede estar relacionado con el hecho de que la población percibe esta medida como protectora, además de ser una acción más directa y fácil de aplicar. Un estudio transversal de 2.013 adultos en EE.UU. y Europa mostró que las motivaciones para el distanciamiento social incluían proteger a todo el mundo (86%), protegerse a uno mismo (84%) y un sentido de responsabilidad para proteger a la comunidad (84%)^(36)^.

Por otro lado, cuatro recomendaciones de protección individual no mostraron cambios significativos a lo largo del tiempo: usar mascarilla al salir de casa, lavarse la mascarilla con agua y jabón al llegar a casa, usar mascarilla en el metro, autobús, tren o coche cuando hay otra persona presente, y desinfectarse las manos al tocar objetos fuera de casa. La falta de una mejora significativa en el uso de la mascarilla al salir de casa puede estar asociada a la elevada puntuación de adherencia registrada desde la primera evaluación, en la que esta medida obtuvo la puntuación más alta. Desde el inicio de la pandemia, el uso de mascarillas ha sido ampliamente promovido y exigido por los organismos públicos como una de las principales medidas de control de la enfermedad, lo que ha redundado en una mayor concienciación y adherencia por parte de la población^(37)^.

Sin embargo, la recomendación con la puntuación más baja desde la primera evaluación fue la de llevar mascarilla en el metro, autobús, tren o coche cuando hubiera otra persona presente. Este resultado puede haberse visto influido por la falta de la alternativa “no aplicable” para los participantes que viajaban a pie o solos en coche, lo que puede haber llevado a muchos a marcar la opción “nunca”, reduciendo la puntuación de esta pregunta. Por lo tanto, este resultado debe interpretarse con cautela.

Un estudio destaca que la pandemia de COVID-19 ha provocado cambios globales en el transporte y en los motivos y la frecuencia de los viajes, con un descenso significativo del volumen de pasajeros del transporte público^(38)^. Un estudio reveló que el 90% de los encuestados había reducido totalmente (47%) o limitado (44%) su uso del transporte público, debido principalmente al cambio de trabajo y/o de clases de modalidad presencial para actividades a distancia, así como al miedo a contraer la enfermedad^(39)^.

El hecho de que no se produjera un aumento significativo del cumplimiento de la obligación de lavarse las mascarillas con agua y jabón al llegar a casa puede atribuirse a la falta de publicidad de esta medida por parte de los organismos gubernamentales y los medios de comunicación. Además, podemos deducir que estos resultados están relacionados con la sobrecarga de trabajo durante el encierro. Un estudio que analizó los retos a los que se enfrentan los trabajadores a distancia durante el cierre identificó dificultades relacionadas con la familia, como el aumento de las responsabilidades escolares, el cuidado de los niños y las tareas domésticas; problemas relacionados con el trabajo, como largas jornadas laborales y espacio inadecuado^(40)^ y problemas de salud mental, como síntomas de ansiedad, depresión y agotamiento^(40-41)^.

La escasa adherencia a la higiene de las manos cuando se tocan objetos fuera del hogar puede explicarse por la falta de acceso o de material adecuado para llevar a cabo esta medida de protección. Un estudio realizado en la ciudad de Woldia, en el noreste de Etiopía, reveló que la mayoría de los participantes (63,1%, n = 255 de 404) no practicaban la higiene de las manos en todos los lugares debido a las restricciones en el acceso al agua y a los productos de higiene^(42)^.

Teniendo en cuenta los resultados obtenidos, los mensajes de texto enviados por teléfono móvil han demostrado ser una herramienta eficaz para la educación en diversos contextos sanitarios, incluida la prevención de enfermedades emergentes. Este enfoque permite llegar a amplios grupos de personas, contribuyendo a la transmisión de información relevante y oportuna y, en consecuencia, mejorando la adherencia al tratamiento y a las medidas de protección.

Los resultados presentados pueden servir de guía a los gestores sanitarios en el desarrollo de programas de prevención y políticas públicas para emergencias sanitarias similares a la pandemia COVID-19. Además, estos resultados pueden ser utilizados para capacitar a los profesionales de la salud, especialmente enfermeras, en el diseño de estrategias de educación sanitaria dirigidas a los grupos más susceptibles a la no adherencia, aumentando la efectividad de las intervenciones y promoviendo una mayor observancia a las medidas de protección.

Este estudio tiene algunas limitaciones. No fue posible comprobar si todos los participantes habían leído los mensajes. Además, al tratarse de una enfermedad emergente, no se disponía de un instrumento validado para evaluar la adherencia a las recomendaciones de protección personal. Teniendo en cuenta la urgencia de las intervenciones para mejorar el conocimiento y el cumplimiento de la población, el instrumento utilizado fue desarrollado por los investigadores del centro principal basándose en las recomendaciones disponibles en ese momento y sólo fue perfeccionado por los demás investigadores del estudio. Además, el instrumento no se probó con la población diana antes de iniciar el estudio, lo que puede haber afectado a algunos de los resultados encontrados y discutidos anteriormente. La pregunta sobre el uso de mascarillas en el transporte público no incluía la alternativa “no procede”, lo que puede haber afectado a los resultados.

En este estudio no se controlaron variables contradictorias como la edad, el nivel educativo, los ingresos y los cambios en las políticas públicas. La falta de control sobre estas variables limita la capacidad de establecer relaciones causales directas entre la intervención y los cambios observados. Por último, la pérdida de muestra observada puede haber afectado a los resultados, limitando la generalización de las conclusiones. Por lo tanto, los efectos identificados deben interpretarse con cautela. A pesar de las limitaciones mencionadas, los resultados son pertinentes y aportan ideas importantes para las políticas públicas y las estrategias de comunicación de salud.

## Conclusión

Los mensajes enviados por teléfono móvil fueron eficaces para promover la adherencia a las prácticas de prevención del COVID-19, especialmente después de la sexta semana de envío. Esta herramienta didáctica podría ser considerada para otras pandemias, debido a su bajo coste y a su probada eficacia para mejorar el conocimiento y la adherencia en diversos contextos.

Los resultados presentados pueden servir de guía para los gestores de salud en el desarrollo de programas de prevención y políticas públicas para emergencias sanitarias similares a la pandemia de COVID-19. Además, el investigador puede utilizar este protocolo para orientar la intervención en futuros estudios. Sin embargo, debido al importante número de pérdidas durante el periodo de seguimiento, se recomienda reducir la duración de este a cuatro semanas.

Además, se recomienda que en futuros estudios se evalúen las pruebas de validez del instrumento desarrollado y se utilice un diseño aleatorio para reforzar aún más los resultados.

## Data Availability

Todos los datos generados o analizados durante este estudio están incluidos en este artículo publicado
